# DNA methylation haplotype block signatures responding to *Staphylococcus aureus* subclinical mastitis and association with production and health traits

**DOI:** 10.1186/s12915-024-01843-y

**Published:** 2024-03-14

**Authors:** Mengqi Wang, Nathalie Bissonnette, Mario Laterrière, Pier-Luc Dudemaine, David Gagné, Jean-Philippe Roy, Marc-André Sirard, Eveline M. Ibeagha-Awemu

**Affiliations:** 1 Sherbrooke Research and Development Centre, Agriculture and Agri-Food Canada, Sherbrooke, QC Canada; 2 Department of Animal Science, Laval University, Quebec, QC Canada; 3 Quebec Research and Development Centre, Agriculture and Agri-Food Canada, Quebec, QC Canada; 4 Department of Clinical Sciences, Université de Montréal, St-Hyacinthe, QC Canada

**Keywords:** Genome-wide DNA methylation alterations, DNA methylation haplotype blocks, Discriminant signatures, Immune functions, Mammary gland health, Milk production, Holstein cow

## Abstract

**Background:**

DNA methylation has been documented to play vital roles in diseases and biological processes. In bovine, little is known about the regulatory roles of DNA methylation alterations on production and health traits, including mastitis.

**Results:**

Here, we employed whole-genome DNA methylation sequencing to profile the DNA methylation patterns of milk somatic cells from sixteen cows with naturally occurring *Staphylococcus aureus* (*S. aureus*) subclinical mastitis and ten healthy control cows. We observed abundant DNA methylation alterations, including 3,356,456 differentially methylated cytosines and 153,783 differential methylation haplotype blocks (dMHBs). The DNA methylation in regulatory regions, including promoters, first exons and first introns, showed global significant negative correlations with gene expression status. We identified 6435 dMHBs located in the regulatory regions of differentially expressed genes and significantly correlated with their corresponding genes, revealing their potential effects on transcriptional activities. Genes harboring DNA methylation alterations were significantly enriched in multiple immune- and disease-related pathways, suggesting the involvement of DNA methylation in regulating host responses to *S. aureus* subclinical mastitis. In addition, we found nine discriminant signatures (differentiates cows with *S. aureus* subclinical mastitis from healthy cows) representing the majority of the DNA methylation variations related to *S. aureus* subclinical mastitis. Validation of seven dMHBs in 200 cows indicated significant associations with mammary gland health (SCC and SCS) and milk production performance (milk yield).

**Conclusions:**

In conclusion, our findings revealed abundant DNA methylation alterations in milk somatic cells that may be involved in regulating mammary gland defense against *S. aureus* infection. Particularly noteworthy is the identification of seven dMHBs showing significant associations with mammary gland health, underscoring their potential as promising epigenetic biomarkers. Overall, our findings on DNA methylation alterations offer novel insights into the regulatory mechanisms of bovine subclinical mastitis, providing further avenues for the development of effective control measures.

**Graphical Abstract:**

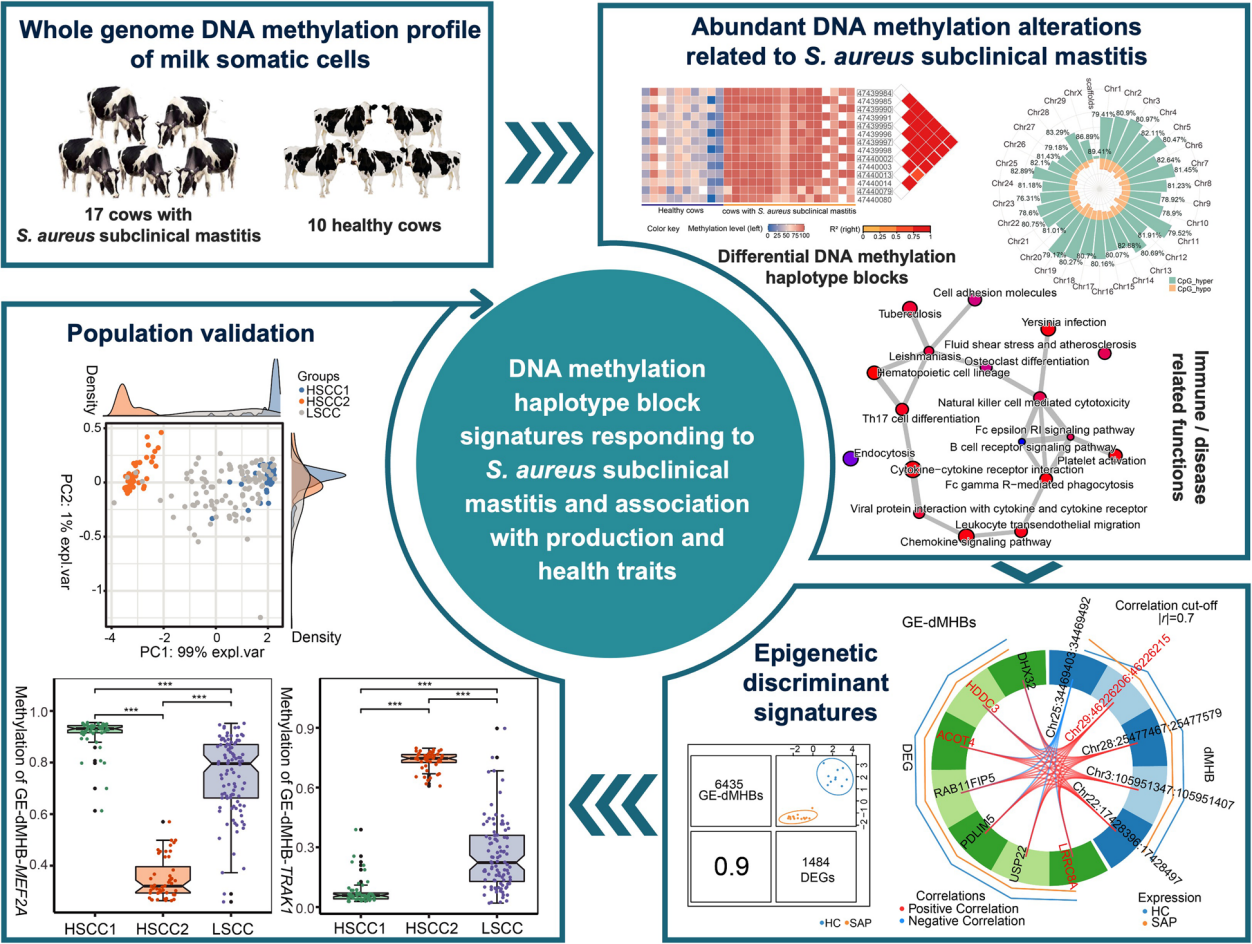

**Supplementary Information:**

The online version contains supplementary material available at 10.1186/s12915-024-01843-y.

## Background

Mastitis, an inflammation of the mammary gland, causes production and economic loses for the dairy industry worldwide. Mastitis also constitutes a major animal welfare problem as well as a potential danger for food and environmental safety, making it a huge challenge for the dairy industry worldwide [1, 2]. Pathogenic bacteria infection is the most common cause of mastitis, with most common infecting species being *Staphylococcus aureus* (*S. aureus*), S*treptococci*, and *Escherichia coli* (*E. coli*). *Staphylococcus aureus* as the most prevalent contagious pathogen can be found on the skin of the mammary gland and teats. In Canada, *S. aureus* is an important causal pathogen of both clinical and subclinical mastitis [3–5]. Mastitis in the subclinical form is without observable symptoms and accounts for a large portion (48%) of the economic costs related to mastitis prevention and control in Canadian dairy farms [6]. Due to its ability to colonize and multiply inside the mammary gland, *S. aureus* subverts host defenses allowing persistent mild subclinical infections characterized by high milk somatic cell count (SCC) [7–9]. It causes irreversible damage to mammary gland tissue resulting in reduced milk production and inducing potential resistance to antibiotics (due to extended application of antibiotics treatments) [10–12].

During the past century, concerted efforts have been directed at developing effective strategies to monitor, prevent, and control mastitis in dairy farms. Farm management strategies and genetic strategies have demonstrated positive contributions towards the improvement of dairy production [1, 13, 14]. Although production traits, conformation and health attributes of farm animals are primarily driven by genetic diversity, it is clear that epigenetic factors, including DNA methylation, constitute an additional important layer influencing these phenotypes. Thus, a plethora of investigations have revealed the impact of epigenetic modifications, including DNA methylation alterations, on animal health and production [15–17] and indications that epigenetic impacts are not fully captured in current conventional genomic breeding programs [17–19]. DNA methylation is one of the most well-characterized epigenetic mechanisms with paramount importance for mammalian development and health, including bovine diseases such as mastitis [15, 19–22].

In recent years, the in vitro infection of bovine mammary epithelial cells with bacterial lipopolysaccharide or *S. aureus* components such as peptidoglycan and lipoteichoic has implicated aberrant DNA methylations in the regulation of immune responses to bovine mastitis [23–25]. Furthermore, the DNA methylation alterations in some immune-related genes, such as *CXCR1*, *IL6R*, *TLR4, NCKAP5*, *CSN1S1*, and *CD4*, have been found to participate in the regulation of gene expression during mastitis caused by *E.coli* or *S. aureus* [26–31]. For instance, hyper-methylation of the promoter region of *CD4* gene was found to negatively correlate with its gene expression changes during mastitis [31–33]. Using deep sequencing technologies, the integration of whole-genome DNA methylation and transcriptome of blood neutrophils identified three genes (*CITED2*, *SLC40A1*, and *LGR4*) as potential candidate markers of *E.coli*-induced mastitis [34]. In addition, the same strategy captured the key changes related to *S. aureus* subclinical mastitis in different tissues, such as mammary gland tissue [35] and peripheral blood lymphocytes in Holstein cows [36]. Collectively, these studies identified thousands of DNA methylation alterations at single cytosine sites or regions on key genes such as *NRG1*, *MST1*, *NAT9*, *IL6R*, *TNF*, *BTK*, *IL1R2*, and *TNFSF8*, indicating the important involvement of DNA methylation in the regulation of the immune response during *S. aureus* subclinical mastitis [34–37]. However, these studies used the RRBS (reduced representation bisulfite sequencing) and the MeDIP-Seq (methylated DNA immunoprecipitation sequencing) methods, which are not able to capture the complete DNA methylation landscape of the whole genome with the consequence that some epigenetic variants were not fully captured.

Most previous studies have made it clear that DNA methylation alterations play important roles in the immune response to mastitis in dairy cows. Thus, the application of more robust technologies (e.g., whole genome methylome sequencing [WGMS]) on large sample numbers will provide knowledge depths on the host regulatory mechanisms, which are crucial for the further practical application of DNA methylation information in controlling mastitis. Therefore, we adopted the WGMS technique and profiled the whole-genome wide DNA methylation patterns of milk somatic cells from 26 Holstein cows, and portrayed the DNA methylation alterations related to *S. aureus* subclinical mastitis in multiple contexts, including single cytosine, methylation haplotype blocks (MHBs), methylation patterns of gene features and repeat elements, and global methylation at the level of the whole genome. We also integrated the DNA methylation alterations and transcriptome data of the same samples [38] to explore their possible biological roles and identify candidate discriminant DNA methylation and gene signatures for *S. aureus* subclinical mastitis. Finally, we validated seven differentially methylated MHBs in 200 cows. Overall, this study has provided a comprehensive resource to better understand the DNA methylation alterations and dynamics during *S. aureus* subclinical mastitis. It also provided candidate discriminant DNA methylation and gene markers that may serve as a catalyst to initiate discussions on the importance of quantifying the effects of epigenetic variations on livestock health and production, and the inclusion of such information in current management and breeding strategies to improve the prediction of breeding values for mastitis resistance.

## Results

### DNA methylation pattern of milk somatic cells

We generated a total of 26 WGMS datasets from milk somatic cells from sixteen cows with *S. aureus* subclinical mastitis and ten healthy control cows (Supplementary Table S1 in Additional file 1). The vast amounts of data (~ 294 million uniquely mapped reads) obtained from each sample revealed ~ 12,215 million cytosines with an average of 27× coverage (ranging from 21.8× to 32.8×) per sample which were used to identify genome-wide DNA methylation cytosine sites (Supplementary Table S2 in Additional file 1). Globally, the average methylation level was 75.28, 0.21, and 0.18% for cytosine in the context of CpG, CHG, and CHH, respectively (where H represents A or T or C). The global DNA methylation level of CpG sites is consistent with data on other somatic tissues in cattle [39], in human [40], and in mouse [41]. Moreover, the global DNA methylation level of CHG sites is consistent with non-CpG methylation recorded in non-brain tissues of cattle (ranged from 0.2 to 0.8%) [39, 42].

**Fig. 1:**
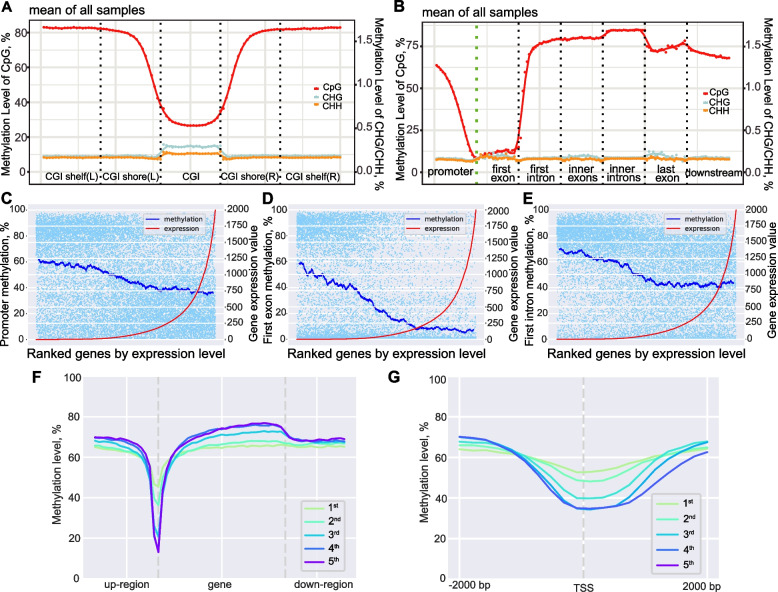
DNA methylation landscape at important genomic functional regions indicating their possible association with gene expression. **A** The distribution of DNA methylation at regions in relation to CpG island (CGI). L: left, R: right. **B** The DNA methylation level at genetic regions illustrating the classic valley-like change of DNA methylation level (CpG sites) around transcription start sites (TSS). **C**–**E** The scatterplot and fitting curves of DNA methylation level of regulatory regions, including promoters (**C**), first exons (**D**), and first introns (**E**), and expression level of genes. The higher the expression level of genes, the lower methylation level of their regulatory regions. **F** The methylation level trends around genes grouped by ranked gene expression level. Up‑region and down-region represent the upstream and downstream region of genes, respectively. **G** An enlarged view of figure **F** near TSS, showing the steeper methylation level trend around TSS for genes with higher expression levels

We also characterized the DNA methylation patterns in specific functional genomic regions to understand their dynamics during *S. aureus* subclinical mastitis. The DNA methylation of CpG sites showed a concave change around CpG island (CGIs) with a downward trend in CGI shores and low level at CGIs (28.74% on average) (Fig. 1A). On the contrary, the CHG and CHH sites showed a slight increase in their methylation levels at CGIs (0.29 and 0.21%, respectively) compared with adjacent regions (Fig. 1A). The methylation level plot among gene features revealed the classic downward trend of CpG sites with a gradual decrease in methylation at the promoter to reach the lowest level in the first exon (Fig. 1B). In addition, CHG and CHH sites showed relatively stable and extremely low methylation level among gene regions, but was slightly higher at exons (Fig. 1B).

We then calculated the average methylation levels of promoters and other gene features for each gene (average value of all qualified CpG sites in corresponding regions) followed by integration analysis of the methylome and transcriptome data with MethGET [43]. The CpG methylation level of promoters showed significant but weak inverse correlation with gene expression level at the genome-wide scale (Pearson’s *R* = − 0.194, *P* = 3.76 × 10^−165^) (Fig. 1C, Supplementary Fig. S1A in Additional file 2), which is consistent with previously reported effects of promoter methylation on transcriptional repression [20, 44]. The general CpG methylation levels of both first exons and first introns also showed significant but weak inverse correlation with gene expression levels at the scale of the whole-genome (Pearson’s *R*: − 0.291 and − 0.173, *P* = 0 and 1.60 × 10^−122^, respectively) (Fig. 1D, E, Supplementary Fig. S1B-C in Additional file 2). This inverse correlation relationship has also been found in human and other model animals which links the methylation at first exon and first intron to possible effects on transcriptional silencing [45, 46]. The valley-like changes of CpG sites at regulatory regions, including promoter, first exon and first intron, were steeper as gene expression levels rise (Fig. 1F), especially around the 2000 bp up- and downstream of transcriptional start sites (TSS) (Fig. 1G). Besides, no significant correlation was found between the gene expression levels and the CpG methylation levels of other gene regions (inner exons, inner introns, and whole gene body) (Supplementary Fig. S1D-F in Additional file 2).

### DNA methylation alterations during *S. aureus* subclinical mastitis

**Fig. 2:**
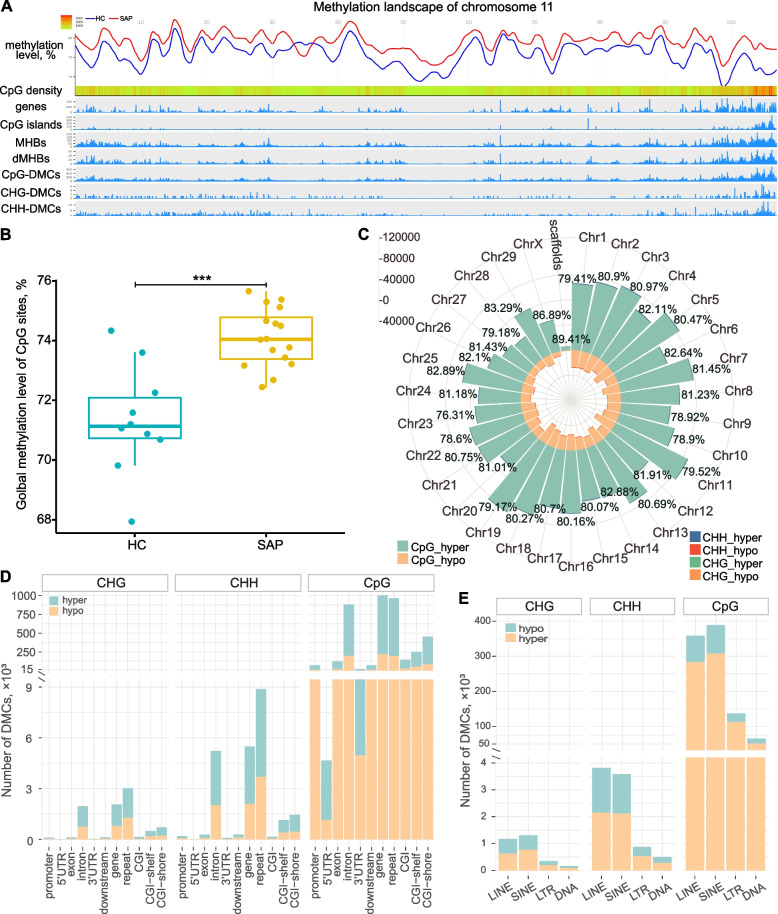
Comparison of DNA methylation status between *S. aureus*-positive (SAP) and healthy control (HC) groups revealed abundant alterations. **A** A DNA methylation landscape of chromosome 11 (Chr11) illustrating the general methylation status and various DNA methylation alterations. A 50-kb-long window was used to count the corresponding information per track. **B** Comparison of global methylation level of CpG sites between SAP and HC groups revealed significantly higher global methylation level of SAP group. ***: significant difference (*P* value = 0.00076). **C** The distribution of DMCs per chromosome (Chr). The number on the top of each bar represents the percentage of hyper-methylated DMCs to total DMCs located in the corresponding Chr. **D** The distribution of DMCs in the context of CpG, CHG, and CHH among genomic functional regions. **E** The count of DMCs collocated in repeat elements, including short and long interspersed retrotransposable elements (SINE and LINE), long terminal repeat retrotransposons (LTR), and DNA transposons (DNA). MHBs: methylation haplotype blocks; dMHBs: differential MHBs; DMCs: differentially methylated cytosines which were counted in the context of CpG, CHG, and CHH, respectively; Hyper/hypo: DMCs were hyper-/hypo-methylated in SAP group compared to HC group; CGI: CpG island. Detailed data on the depicted findings are found in Supplementary Tables 3–6

We compared the DNA methylation of cytosine sites between *S. aureus*-positive (SAP) and healthy control (HC) groups at different layers to investigate the DNA methylation alterations during *S. aureus* subclinical mastitis. Firstly, the global methylation level of CpG sites of SAP group was significantly higher than HC group at a scope of each chromosome (Chr) (Fig. 2A shows Chr11 only, Supplementary Table S3A in Additional file 1) as well as whole genome (Fig. 2B, Supplementary Table S3B in Additional file 1) (FDR < 0.05). This significant higher methylation level of CpG sites in SAP group was also seen at genomic functional regions, including gene features, CGIs, CGI shores, and CGI shelves (Supplementary Table S3C in Additional file 1, Supplementary Figs. S2-S3 in Additional file 2). However, the difference in methylation levels of CHG and CHH sites between SAP and HC group was not significant at genome-wide scale (Supplementary Fig. S4 in Additional file 2), as well as in most genomic functional regions (FDR > 0.05) (Supplementary Table S3C in Additional file 1) except CHG sites which showed significantly higher expression in SAP group at CGI (FDR = 0.025) (Supplementary Fig. S5 in Additional file 2).

Furthermore, we compared the methylation of each cytosine between SAP and HC group using MethylKit [47], and identified a total of 3,328,843, 7255, and 20,358 differentially methylated cytosine sites (DMCs) in the context of CpG, CHG, and CHH, respectively (*q*-value < 0.05 and |methylation difference|> 20%) (Supplementary Tables S4-S5 in Additional file 1). Consistent with the globally higher methylation of SAP group, 80.73% of CpG-DMCs and 61.6% of CHG- and CHH-DMCs were hyper-methylated in SAP group compared with HC group (*q*-value < 0.05, methylation difference ≥ 20%) (Fig. 2C). At Chr scale, Chr11 harbored the most DMCs (*n* = 162,144, 4.83%), followed by Chr19 (*n* = 157,932, 4.70%) and Chr5 (*n* = 155,308, 4.62%), while the least DMCs (*n* = 56,880, 1.69%) were found on Chr28 (Fig. 2C, Supplementary Table S6A in Additional file 1). The annotation of DMCs to gene features revealed that 69% DMCs were located in intergenic regions (Supplementary Table S6B in Additional file 1). Also, 1,148,481 CpG-, 2295 CHG-, and 5987 CHH-DMCs were located in genes and their 2 kb upstream (promoters) and 2 kb downstream regions. Out of these, more DMCs were located in introns (*n* = 887,913, 76.76%) followed by exons (*n* = 112,914, 9.76%) (Fig. 2D). Besides, we found 72,855 (6.30%), 11,599 (1.00%), and 308,719 (26.69%) DMCs in promoters, first exons, and first introns, respectively. 3′UTR regions harbored more DMCs than 5′UTR. However, 5′UTR had the highest density of DMCs (6.15 DMCs/100 bp), followed by first exon (2.52 DMCs/100 bp) and 3′UTR (1.90 DMCs/100 bp) (Supplementary Table S7 in Additional file 1).

On the other hand, we identified a total of 13,358 genes harboring at least 1 DMC in any of three contexts, and 76.58% of them (*n* = 10,230) harbored greater than 20 DMCs (Supplementary Table S7 in Additional file 1). Integration with transcriptome data revealed that 3599 out of 13,358 genes harboring DMCs were identified as differentially expressed genes (DEGs) between SAP and HC group (|log_2_FC|> 1 and FDR < 0.05). The DEGs harboring DMCs (*n* = 3599) were significantly enriched in 20 gene ontology (GO) terms and 35 Kyoto Encyclopedia of Genes and Genomes (KEGG) pathways mostly having immune- or disease-related functions (Supplementary Table S8A in Additional file 1). Interestingly, DEGs harboring DMCs at their first exon (*n* = 431) were significantly enriched in immune-related KEGG pathways, including cytokine-cytokine receptor interaction (bta04060), *Staphylococcus aureus* infection (bta05150), and viral protein interaction with cytokine and cytokine receptor (bta04061) (Supplementary Table S8B in Additional file 1). In addition to immune- or disease-related functional annotations, DEGs harboring DMCs in the promoter region (*n* = 2735) were also enriched in KEGG pathways related to metabolism processes, such as fatty acid metabolism (bta01212), propanoate metabolism (bta00640), and fatty acid degradation (bta00071) (Supplementary Table S8C in Additional file 1). This observation suggests that the methylation alterations may be involved in the regulation of mammary gland responses during *S. aureus* subclinical mastitis, and the DNA methylation at different genetic regions may play different roles.

### DNA methylation alterations of repeat elements in response to *S. aureus* subclinical mastitis

**Fig. 3:**
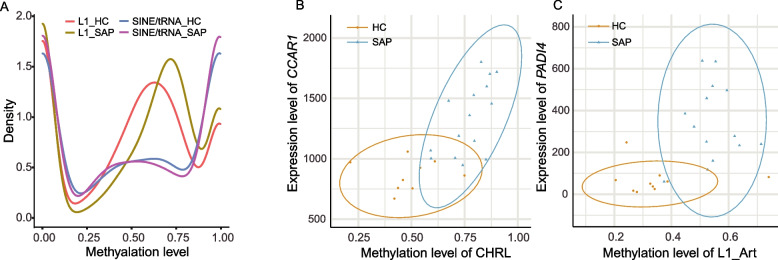
Comparison of the methylation levels of repeat elements between *S. aureus*-positive (SAP) and healthy control (HC) groups. **A** The methylation level distribution of LINE-1 (L1) and tRNA-derived SINE (SINE/tRNA) in SAP and HC groups. **B**, **C** The methylation level of CHRL (a tRNA-derived SINE, Chr28:25046248-25046370) (**B**) and L1-Art (a LINE-1, Chr2:135290744-135291147) (**C**) and the expression level of their overlapped genes, *CCAR1* and *PADI4*, per sample, revealing significant differences between SAP and HC groups as well as positive correlation between methylation level and expression level of their overlapped genes. Detailed data on the depicted findings are found in Supplementary Table 9

Among all identified DMCs, we found that 28.96% CpG-DMCs (964,144), 41.60% CHG-DMCs (3018), and 43.63% CHH-DMCs (8883) were located in repeat elements (REs) (Fig. 2D). The DMCs were mainly located in non-long terminal repeat retrotransposons, including short interspersed nuclear elements (SINEs) and long interspersed nuclear elements (LINEs), followed by long terminal repeat (LTR) retrotransposons and DNA transposons (Fig. 2E). To further explore the DNA methylation status of REs and their possible changes during *S. aureus* subclinical mastitis, we used a random forest-based algorithm to calculate the methylation levels of LINE (LINE-1 elements) and SINE (tRNA-derived SINEs), which are the most abundant retrotransposons in the bovine genome. After filtering, a total of 321 LINE-1 elements and 217 tRNA-derived SINEs harboring at least two CpG sites were retained (Supplementary Table S9A-B in Additional file 1). In general, tRNA-derived SINEs had higher CpG density (2.22 CpG/100bp) than LINE-1 elements (0.94 CpG/100bp) (Supplementary Fig. S6 in Additional file 2). However, LINE-1 elements had generally a higher methylation level than tRNA-derived SINEs; their median methylation levels were 60 and 55%, respectively. As shown in Fig. 3A, LINE-1 elements in SAP group showed clearly higher density and methylation level (> 60%) compared to HC. In addition, 96 LINE-1 elements and 23 tRNA-derived SINEs showed significant difference in their methylation levels between SAP and HC group (FDR < 0.05), which also correlated significantly with *S. aureus* subclinical mastitis status (FDR < 0.05 and |c_pb_|> 0.3) (Supplementary Table S9c in Additional file 1). Among them, twelve LINE-1 elements and nine tRNA-derived SINEs were collocated with protein-coding genes, and the collocated genes of one LINE-1 (L1_Art, Chr2:135290744-135291147) and three tRNA-derived SINEs (CHR-2A (Chr1:64176302-64176611), CHRL (Chr28:25046248-25046370), and CHR-2B (Chr25:21749299-21749545)) were significantly differentially expressed between SAP and HC group. The methylation status of L1_Art, CHR-2A, and CHRL showed significantly strong and positive correlation with the expression levels of their overlapped genes (*PADI4*, *ARHGAP31*, and *CCAR1*, respectively) (Fig. 3B, C). It is worth noting that L1_Art and CHRL harbored the TSS of *PADI4* and *CCAR1*, respectively. This suggests that the methylation changes of these REs, such as L1_Art, CHR-2A, and CHRL, may be a possible mechanism underlying the gene expression changes of their overlapped genes during *S. aureus* subclinical mastitis.

### Methylation haplotype blocks responding to *S. aureus* subclinical mastitis

To better identify small regions with abundant DNA methylation alterations, we used MONOD2 to identify MHBs that captures the co-methylation status of adjacent CpG sites exhibiting highly coordinated methylation [48]. Using a linkage disequilibrium (*r*^2^) cutoff of 0.5 and minimum 3 CpG sites per block, we identified a total of 750,583 MHBs. The majority of CpG sites within the same MHB were tightly coupled (*r*^2^ ~ 1). The MHBs had a median length of 33.0 bp (range from 6 to 388 bp) and a median CpG density of 10.34 CpG/100 bp, which represents about 0.91% of the bovine genome (Supplementary Fig. S7A-B in Additional file 2). About three quarters of MHBs (576,014) were located in the intergenic region, while about 40% (294,672) were overlapped with transcripts. MHBs were also widely distributed in known regulatory regions, such as promoters, CGIs, exons, and introns (Supplementary Fig. S7C in Additional file 2). The overlapping of MHBs with different genomic functional regions suggests their potential to represent a distinct type of genomic feature.

**Fig. 4:**
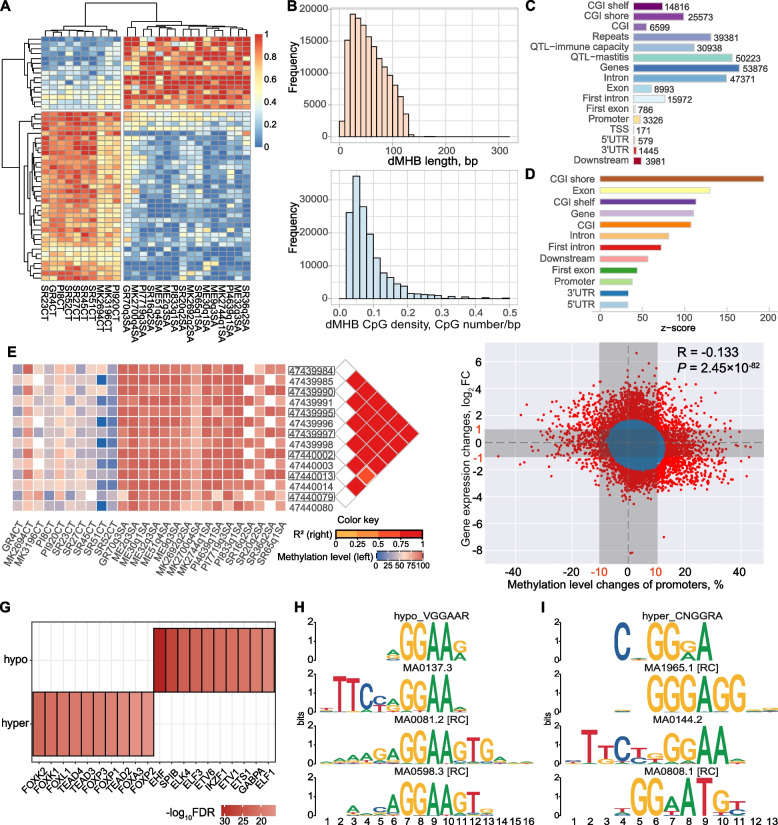
The dMHBs and the potential effects of DNA methylation at regulatory regions on gene expression. **A** Heap map of top 50 most variable dMHBs. **B** The length and CpG density distribution of dMHBs. **C** Co-localization of dMHBs with genomic functional regions. CGI: CpG islands. **D** Enrichment of dMHBs at genomic functional regions. **E** An example of dMHB (Chr29:47439984:47440080) showing the coordinated methylation of CpG sites in the same dMHB. **F** Global relationship (significant negative correlation) between promoter methylation level and gene expression level. Each dot symbolizes a specific gene. Blue dots indicate that gene expression and methylation level changes for the corresponding gene were not statistically significant (Gaussian Mixture Model *p* > 0.005). Conversely, red dots represent differential genes with significant changes in both gene expression and methylation levels of their promoters (Gaussian Mixture Model *p* < 0.005). Red dots out of gray shadow represent differential genes with significant changes in the methylation level of promoter (greater than 10% changes) and gene expression level (|log_2_FC|≥ 1) between *S. aureus*-positive (SAP) and healthy control (HC) groups. **G** The top 10 most significantly enriched known motifs for transcription factors in hyper- and hypo-methylated dMHBs located at regulatory regions and significantly associated with gene expression (GE-dMHBs). **H**, **I** Examples of de novo (discovered) motifs in hypo-methylated GE-dMHBs (hypo_VGGAAR) (**H**) and hyper-methylated GE-dMHBs (hyper_CNGGRA) (**I**), showing high similarity with known motifs for transcription factors. Detailed data on the depicted findings are found in Supplementary Tables 10, 11 and 14

To allow the direct comparison of the methylation status of MHBs between groups, we calculated the methylation haplotype load (MHL) for each MHB which was defined as the normalized fraction of methylation haplotypes at different lengths [48]. We found a total of 153,783 MHBs with significant differences between SAP and HC groups (|MHL difference|> 20% and FDR < 0.05), and are here referred to as differential MHBs (dMHBs) (Supplementary Table S10A in Additional file 1). Among them, 120,033 dMHBs (78.05%) showed higher methylation levels in SAP group. The dMHBs were able to capture clearly the initial classification of SAP and HC groups, as exampled by the most variable dMHBs in Fig. 4A. The length of dMHBs, ranged from 6 to 318 bp, with median length of 53 bp and median CpG density of 6.90 CpG/100 bp (Fig. 4B). The dMHBs harbored a total of 624,957 CpG-DMCs, accounting for 18.77% of total CpG-DMCs. The dMHBs showed similar distribution characteristics with DMCs. The majority of dMHBs (100,141, 65.12%) were found in intergenic regions while 53,876 dMHBs were overlapped with genes (Fig. 4C). The dMHBs also showed significant enrichment in functional regulatory and genic regions, such as exons, introns, first intron, downstream region, first exon, promoter, 3′UTR, and 5′UTR (*P* value < 0.001) (Fig. 4D). In addition, 30,938 and 50,223 dMHBs were collocated with QTLs for immune capacity and mastitis, respectively (Fig. 4C). It is worth noting that some QTLs coved a very large region and harbored many dMHBs, such as a QTL (#10446, Supplementary Table S10B in Additional file 1) for somatic cell score, which is about 61.3 Mbp long and harbors 8436 dMHBs. Since the extremely long QTLs are usually identified by linkage methods using low-density microsatellites marker maps, we further checked the QTLs with less than one Mbp length and found 73 immune capacity QTLs and 181 mastitis QTLs harboring 355 and 1334 dMHBs, respectively (Supplementary Table S10B in Additional file 1). Among them, 13 dMHBs harbored SNP markers, including three, two, seven, and one dMHBs harboring SNPs for clinical mastitis, SCC, somatic cell score, and eosinophil number, respectively. For instance, Chr1:110158892:110158992 which collocated with exon 3 of *PTX3* harbored two SNPs for somatic cell score (QTL #219865 and #219866). Besides, most of the CpG sites in the same dMHB were tightly linked DMCs (*r*^2^ ~ 1) which also showed clear differences in their methylation levels between SAP and HC groups (Fig. 4E).

### DNA methylation at regulatory regions as potential regulators of gene expression

We found significant but weak inverse correlation between the changes in global CpG methylation levels of regulatory regions, including promoter, first exon, and first intron, and corresponding gene expression level changes between SAP and HC groups at genome-wide scale (*P* < 5 × 10^−8^) (Fig. 4F, Supplementary Fig. S8 in Additional file 2). Additionally, we identified 882, 1188, and 870 genes with significant changes in their gene expression levels and CpG methylation levels of promoter, first exon, and first intron, respectively, and here referred to as differential genes (|log_2_FC|> 1, |CpG methylation difference|> 10% and *P* value < 0.005) (Supplementary Table S11 in Additional file 1). The differential genes were significantly enriched in GO terms and KEGG pathways related to key functions of immune responses and diseases, such as *Staphylococcus aureus* infection (bta05150), antigen processing and presentation (bta04612), cytokine-cytokine receptor interaction (bta04060), positive regulation of immune system process (GO:0002684) among others, suggesting the potential involvement of DNA methylation at these regulatory regions in the host response to defense against *S. aureus* infection (Supplementary Table S12 in Additional file 1). Interestingly, the CpG methylation level of the regulatory regions of more than 50% of the differential genes (promoter: 58.28%, first exon: 50.84%, first intron: 66.09%) were significantly correlated their gene expression levels (|Spearman’s rho|> 0.3 and FDR < 0.05, Supplementary Table S11 in Additional file 1). In particular, majority of them had hyper-methylated promoters (*n* = 286), first exons (*n* = 300), or first introns (*n* = 347) which were significantly negatively correlated with the downregulated expression of their corresponding genes. This further revealed that DNA methylation in regulatory regions may mediate gene repression to regulate the immune responses of the mammary gland during *S. aureus* subclinical mastitis.

Therefore, we selected the dMHBs that overlapped with promoters (*n* = 1261, pro-dMHBs), first exons and 5′UTR (*n* = 526, FE-dMHBs), and first introns (*n* = 6921, FI-dMHBs) of DEGs to further investigate their possible regulatory roles in gene expression during *S. aureus* subclinical mastitis. We used Spearman’s rank correlation coefficient (rho) to correlate each dMHB to its corresponding DEG. We found that the methylation status of 6435 dMHBs (933 pro-, 400 FE-, and 5250 FI-dMHBs) were significantly correlated with the expression levels of their corresponding DEGs (|rho|> 0.3 and FDR < 0.05), here referred to as gene expression correlated dMHBs (GE-dMHBs) (Supplementary Table S13 in Additional file 1). Among them, the majority of GE-dMHBs, including 76.85% of pro-GE-dMHBs (*n* = 717), 85.50% of FE-GE-dMHBs (*n* = 342), and 71.37% of FI-GE-dMHBs (*n* = 3747) had significant negative correlations between their methylation status and the expression levels of their corresponding DEGs (rho < − 0.3 and FDR < 0.05). In particular, the negatively correlated hyper-methylated GE-dMHBs and their corresponding downregulated DEGs accounted for the largest proportion (i.e., 533 pro-, 137 FE-, and 627 FI-GE-dMHBs).

To explore the possible effects of GE-dMHBs on transcriptional factor (TF) binding regions, we performed a motif search of expressed TFs in 6435 GE-dMHBs, including 4999 hyper- and 1436 hypo-methylated GE-dMHBs. Firstly, 144 and 67 known motifs were significantly enriched in hyper- and hypo-methylated GE-dMHBs, respectively (FDR < 0.05) (Fig. 4G, Supplementary Table S14A in Additional file 1). The most significantly enriched motifs, such as the Fork head/winged helix factors (FOXK1, FOXK2, FOXL1, FOXP1, FOXA3, and others), the TEA domain factors (TEAD1-TEAD4), and the tryptophan cluster factors (EHF, ETV1, ETV4, ELF1, ELF3, and others), have been implicated in the regulation of the expression of a wide range of genes [49, 50]. The enriched SP4, EGR1, and KLF4 TF motifs have been found to regulate gene expression through binding to CpG-rich promoters [51, 52]. Additionally, the discovery of de novo motifs by running Dreme from MEMEs [53] revealed 23 and 13 novel motifs within hyper- and hypo-methylated GE-dMHBs, respectively (Supplementary S14B-C, Supplementary Fig. S9 in Additional file 2). For instance, hypo_VGGAAR and hyper_CNGGRA, the most significantly enriched motifs discovered in hypo- and hyper-methylated GE-dMHBs, respectively, showed high similarity with known STAT domain factors (STAT1 and STAT3, respectively) (Fig. 4H, I). Therefore, the enrichment of known and de novo TF motifs in GE-dMHBs suggests the potential effects of GE-dMHBs on TF binding events and thereby transcriptional activities.

### Potential functions of GE-dMHBs in response to *S. aureus* subclinical mastitis

**Fig. 5:**
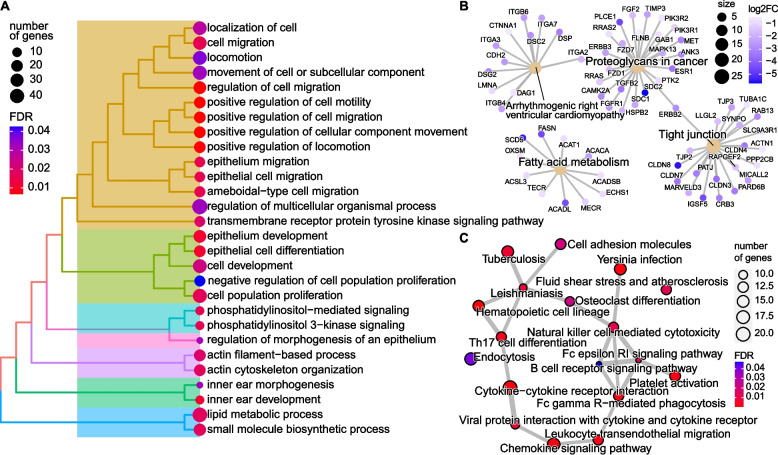
Functional enrichment for gene expression associated differential methylation haplotype blocks (GE-dMHBs). **A**, **B** Tree-plot showing the clustering of biological process GO terms (**A**) and enrichment map of four KEGG pathways (**B**), which were significantly enriched by downregulated differentially expressed genes (DEGs) that were negatively correlated with their overlapped hyper-methylated GE-dMHBs. **C** Enrichment map of 18 KEGG pathways significantly enriched by upregulated DEGs that negatively correlated with their overlapped hypo-methylated GE-dMHBs. Detailed data on the depicted findings are found in Supplementary Table 15

We applied functional enrichment analysis to GE-dMHBs (their overlapped and significantly correlated DEGs) to explore their potential roles during *S. aureus* subclinical mastitis using clusterProfiler [54]. GE-dMHBs that negatively correlated with their overlapped DEGs were enriched in more functional GO terms and KEGG pathways than those with positive correlations. Among them, 3604 hyper-methylated GE-dMHBs (they were negatively correlated with their overlapped 816 downregulated DEGs) were significantly enriched in 38 GO terms (28 biological process (BP) (Fig. 5A), one molecule function (MF) and nine cellular component (CC)), and four KEGG pathways (Fig. 5B) (Supplementary Table S15A in Additional file 1). As showed in Fig. 5A, the BP-GO terms were classified into clusters according to their pairwise similarities. The biggest cluster of BP-GO terms are involved in cell migration, locomotion, and related regulation, reflected through most significantly enriched BP-GO terms like positive regulation of cell migration (GO:0030335, FDR = 0.006), positive regulation of cell motility (GO:2000147, FDR = 0.006), and regulation of cell migration (GO:0030334, FDR = 0.006) among others (Supplementary Table S15A in Additional file 1). The second cluster of BP-GO terms are related to cell development, such as cell population proliferation (GO:0008283, FDR = 0.02), epithelial cell differentiation (GO:0030855, FDR = 0.01), and epithelium development (GO:0060429, FDR = 0.01), which are important for maintenance of mammary gland health. In addition, two BP-GO terms related to metabolic processes were enriched and clustered together, including lipid metabolic process (GO:0006629, FDR = 0.02) and small molecule biosynthetic process (GO:0044283, FDR = 0.02). Meanwhile, a KEGG pathway related to lipid metabolism, the fatty acid metabolism (bta01212, FDR = 0.03), was also enriched (Fig. 5B). This suggests that the hyper-methylated GE-dMHBs may participate in regulating mammary gland health and biological functions, such as cell migration, cell development, and metabolic processes, by mediating the repression of related genes during *S. aureus* subclinical mastitis. Furthermore, a total of 32 downregulated DEGs that negatively correlated with their overlapped hyper-methylated GE-dMHBs, such as *ACTN1*, *CLDN3*, *CLDN4*, *CXCL17*, *CXADR*, *FGF1*, *FGF2*, *TGFB2*, *SPRY2*, and *SLC9A3R1*, among others, were enriched in at least 10 GO terms and/or KEGG pathways related to the functions mentioned above, highlighting their important roles in mammary gland health (Supplementary Table S15B in Additional file 1).

Besides, the 1075 hypo-methylated GE-dMHBs (negatively correlated with their overlapped 369 upregulated DEGs) were significantly enriched in 18 KEGG pathways, mostly immune and disease-related terms (Fig. 5C, Supplementary Table S15A in Additional file 1). For instance, some key functions of immune defense process were significantly enriched, including chemokine signaling pathway (bta04062, FDR = 0.002), cytokine-cytokine receptor interaction (bta04060, FDR = 0.002), leukocyte transendothelial migration (bta04670, FDR = 0.002), Th17 cell differentiation (bta04659, FDR = 0.004), natural killer cell-mediated cytotoxicity (bta04650, FDR = 0.011), and B cell receptor signaling pathway (bta04662, FDR = 0.047), among others (Fig. 5C). It is worth mentioning that 16 upregulated DEGs with hypo-methylated GE-dMHBs in their regulatory regions, including *RAC2*, *IL1B*, *IL2RA*, *BOLA-DOB*, *SYK*, and *NCF1*, among others, were enriched in five or more of the immune/disease-related KEGG pathways (Supplementary Table S15B in Additional file 1). These enriched KEGG pathways indicated that the absence or scarcity of DNA methylation in these GE-dMHBs may be a possible regulatory mechanism underlying the upregulated expression of related genes and potential regulation of the immune defense of mammary gland against *S. aureus* invasion. On the other hand, the GE-dMHBs that positively correlated (FDR < 0.05 and rho > 0.3) with their overlapped DEGs accounted for a small part (27%) of the GE-dMHBs and were enriched in few GO terms or KEGG pathways. Upregulated DEGs (*n* = 386) with correlated hyper-methylated GE-dMHBs (*n* = 1399) were enriched in three CC-GO terms and five KEGG pathways (Supplementary Table S15A in Additional file 1). Meanwhile, the 187 downregulated DEGs with correlated hypo-methylated dMHBs (*n* = 364) were enriched in four CC-GO terms only (Supplementary Table S15A in Additional file 1). Furthermore, we also submitted for functional enrichment the DEGs that correlated with their overlapped pro-, FE-, and FI-GE-dMHBs separately. In general, the functional annotations enriched by pro-, FE-, and FI-GE-dMHBs were similar with those of all GE-dMHBs (Supplementary Table S15C-E in Additional file 1, Fig. S10 in Additional file 2).

### Identification of discriminant signatures from GE-dMHBs

**Fig. 6:**
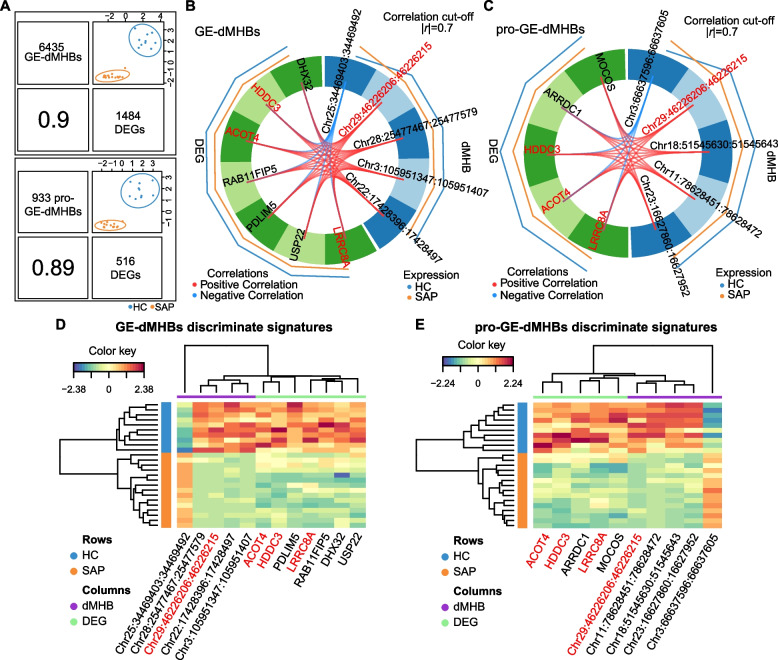
Candidate discriminant signatures related to *S. aureus* subclinical mastitis. **A** The plot at the top is based on all GE-dMHBs that collocated at regulatory regions and significantly correlated with their overlapped differentially expressed genes (DEGs). The plot at the bottom shows only GE-dMHBs that overlapped with promoters (pro-GE-dMHBs) and their corresponding DEGs. For each plot, the upper diagonal represents the first component and the lower diagonal the Pearson correlation between each component. **B**, **C** Circos plot showing the correlation between discriminant signatures from the data set of all GE-dMHBs (**B**) and only pro-GE-dMHBs (**C**). Positive (*r* > 0.7) and negative (*r* < -0.7) correlations are indicated by red and blue links, respectively. The external lines display the relative expression/methylation levels of discriminant signatures with respect to each outcome category. The yellow and blue lines represent the gene expression/methylation levels of *S. aureus*‑positive (SAP) and healthy control (HC) groups, respectively, and the outer line represents the higher level. **D**, **E** Clustered Image Map (Euclidean distance, Complete linkage) of the discriminant signatures (GE-dMHBs and DEGs) from the data set of all GE-dMHBs (**D**) and only pro-GE-dMHBs (**E**). Samples are displayed in rows and discriminant signatures in columns. Detailed data on the depicted findings are found in Supplementary Table 16

We used DIABLO method to identify a subset of discriminant and highly correlated signatures across different omics by integrating the 6435 GE-dMHBs and their overlapped correlated DEGs (*n* = 1484). Firstly, the latent component of the two OMICs (GE-dMHBs and DEGs) were highly correlated (*r* = 0.90, Fig. 6A). After finetuning, five GE-dMHBs, including one pro-GE-dMHBs and four FI-GE-dMHBs, and seven DEGs discriminant signatures were found to explain the most variations delineating the SAP and HC cows (Fig. 6B–E, Supplementary Table S16 in Additional file 1). Although the discriminant GE-dMHBs were not directly located in the discriminant DEGs, they were significantly and strongly correlated with each other (Fig. 6B). The only pro-GE-dMHB, Chr29:46226206:46226215 located in the promoter of *CPT1A*, was the most important with highest loading weight (0.79). FI-GE-dMHB, Chr28:25477467:25477579 located in the first intron of *SRGN*, was the second most important (loading weight = 0.51). These two GE-dMHBs and the fourth discriminant signature, Chr3:105951347:105951407 located in the first intron of *RLF*, were all hypo-methylated in SAP group and strongly negatively correlated with the upregulated expression of their overlapped DEGs. The fifth signature, Chr22:17428396:17428497 located in the first intron of *SRGAP*3, showed positive correlation between its hypo-methylation and the downregulated expression of *SRGAP*3 in SAP group. While the third signature, Chr25:34469403:34469492 located in the fist intron of *DTX2*, was hyper-methylated in SAP group and positively correlated with the upregulated expression of *DTX2*. However, all seven DEGs were downregulated in SAP group. Among them, three (*ACOT4*, *PDLIM5*, and *RAB11FIP5)* were also selected as discriminant genes driving the major changes in gene expression profiles of key gene modules related to *S. aureus* subclinical mastitis in our previous transcriptome analysis [38].

Many studies have demonstrated the significant effects of DNA methylation in promoter region on transcriptional activities, especially gene expression repression [55–57]. Therefore, we further summited 933 pro-GE-dMHBs and 516 overlapped DEGs to DIABLO method and identified ten signatures, including five pro-GE-dMHBs and five DEGs (Fig. 6C, Supplementary Table S16 in Additional file 1). Notably, the first signature (Chr29:46226206:46226215) with highest importance (loading weight = 0.93) was also identified as the most important signature based on all GE-dMHBs, further highlighting its capability to discriminate SAP group from HC group. In addition, four pro-GE-dMHBs were also selected as discriminant signatures but with lower loading weight, including Chr3:66637596:66637605, Chr18:51545630:51545643, Chr23:16627860:16627952, and Chr11:78628451:78628472 located in the promoters of *GIPC2*, *CD177*, *PPP2R5D*, and *SDC1*, respectively. The second (Chr3:66637596:66637605) and third (Chr18:51545630:51545643) signatures are hyper- and hypo-methylated in SAP group respectively, and also negatively correlated with their overlapped DEGs. While the last two signatures (Chr23:16627860:16627952 and Chr11:78628451:78628472) were both hypo-methylated and positively correlated with the downregulated expression of their overlapped DEGs. Moreover, all five discriminant DEGs were also downregulated in SAP group. Besides, three DGEs (*ACOT4*, *HDCC3*, and *LRRC8A*) were also identified in both analyses. As showed in Fig. 6D, E, the discriminant GE-dMHBs and DEGs displayed a nice classification of SAP and HC group, suggesting their potential to be candidate discriminant markers for *S. aureus* subclinical mastitis that needs further validation in larger herds.

### Validation of the DNA methylation status of seven GE-dMHBs in a larger sample size

**Fig. 7:**
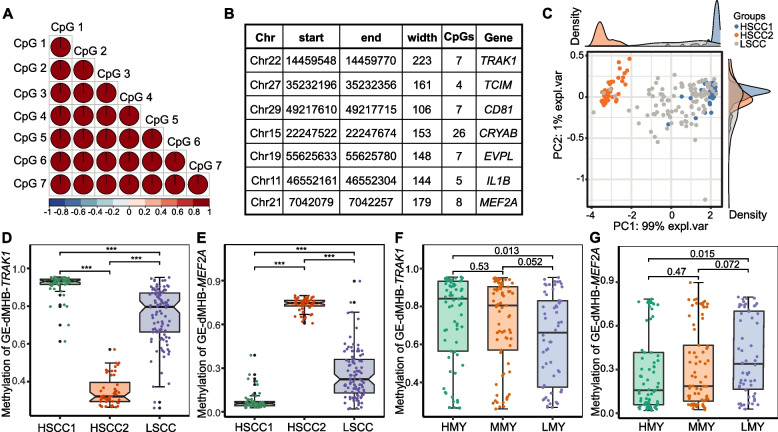
Validation of the DNA methylation status of seven GE-dMHBs in 200 cows. **A** Correlation plot of GE-dMHBs located in the first exon of *TRAK1* (GE-dMHBs-*TRAK1*) as example, showing strong positive correlation between CpG sites detected in GE-dMHBs. **B** List of seven GE-dMHBs subjected to targeted bisulfite amplicon sequencing in 200 cows. **C** Principal component analysis plot illustrating the clustering of cows with HSCC into two sub-groups (HSCC1 and HSCC2) with extremely different methylation status of the GE-dMHBs. **D**, **E** Boxplot showing the significantly different methylation status of GE-dMHBs-*TRAK1* (**D**) and GE-dMHBs-*MEF2A* (**E**) between three groups. *** denotes a statistically significant difference between the corresponding groups, with *p* < 0.05. **F**, **G** Boxplot showing the significantly different methylation status of GE-dMHBs-*TRAK1* (**F**) and GE-dMHBs-*MEF2A* (**G**) between cows with different milk yield, including high (HMY, ≥ 40 kg/day, *n* = 69), middle (MMY, 30 ~ 40 kg/day, *n* = 73), and low milk yield (LMY, ≤ 30 kg/day, *n* = 58). The number atop each bar represents the *p*-value between the respective groups. Detailed data on the depicted findings are found in Supplementary Table 17

To validate the methylation status of GE-dMHBs and explore their possible practical applications, we used targeted bisulfite amplicon sequencing (TBAS) to detect the methylation status of some GE-dMHBs in milk somatic cells in a larger sample size (200 cows from nine herds and do not include individuals used in the discovery phase) (Supplementary Table S17A in Additional file 1). Unfortunately, primer selection for the discriminant signatures identified by DIABLO above failed. Therefore, we considered seven GE-dMHBs that passed primer design criteria, including two hypo-methylated GE-dMHBs found in the promoter regions of *IL1B* and *MEF2A*, and five hyper-methylated GE-dMHBs found in the first exon of *TRAK1* and the promoter regions of *CD81*, *CRYAB*, *EVPL*, and *TCIM*. Two-hundred cows were grouped according to their SCC records from dairy herd improvement (DHI) test results into high SCC group (HSCC, *n* = 100) and low SCC group (LSCC, *n* = 100) (Supplementary Table S17B in Additional file 1). Then, genomic DNA isolated from milk somatic cells of these 200 cows were used for TBAS of seven GE-dMHBs. Following PCR amplification and library preparation, the seven GE-dMHBs were sequenced with at least ten thousand reads coverage depth per CpG site per cow to further explore their methylation status (Supplementary Table S17C-D in Additional file 1). We firstly checked the correlation between detected CpG sites per GE-dMHB, which indicated that all of them were significantly and strongly correlated (*r* > 0.8), supporting the co-methylation status of adjacent CpG sites in MHBs (Fig. 7A, Supplementary Table S17E in Additional file 1). In addition, TBAS data identified significant correlation between neighboring CpG sites surrounding GE-dMHBs and CpG sites inside GE-dMHBs, suggesting that deeper sequencing with larger sample size may help to improve the identification of MHBs with more co-methylated CpG sites (Supplementary Table S17E in Additional file 1). Then, the methylation levels of each GE-dMHB per sample were calculated as the arithmetic mean of CpG sites they harbored as well as the correlated neighboring CpG sites (Fig. 7B). Moreover, as shown in Fig. 7C, the methylation status of the GE-dMHBs showed obvious clustering for cows with HSCC and LSCC. In particular, cows of HSCC group were further clustered into two sub-groups (HSCC1 [mean SCC = 663,903 cells/mL] and HSCC2 [mean SCC = 734,291 cells/mL]), whose GE-dMHBs had extremely different methylation levels. Given the absence of a significant batch effect (parity, lactation stage, and farm) in the background information of cows, the observed division in the methylation status of HSCC cows prompts further investigation. For example, the high SCC of cows in the HSCC group might have been caused by the presence of different pathogens or factors. Additionally, variations in infection stages or the duration of infection could potentially contribute to the observed disparities in methylation status. Therefore, we divided HSCC group into HSCC1 and HSCC2 according to PCA clustering for the statistical comparisons.

The comparison of methylation status of the seven GE-dMHBs revealed significant differences between HSCC1, HSCC2, and LSCC groups (FDR < 0.05) (Supplementary Table S17F in Additional file 1). Taking the hyper-methylated GE-dMHB in the first exon of *TRAK1* (GE-dMHBs-*TRAK1*) as an example (Fig. 7D), GE-dMHB-*TRAK1* had significantly different methylation levels between the three groups with highest level in HSCC1 (91.15%) and lowest level in HSCC2 (34.97%). Interestingly, the methylation level changes of GE-dMHB-*TRAK1* in HSCC1 group compared to LSCC group (16.30%) was similar with its change between SAP and HC cows identified by WGMS data (hyper), while the methylation level changes in HSCC2 group compared to LSCC group (− 39.88%) was the opposite of that identified by WGMS data. Moreover, the similar methylation changes in HSCC1 group and opposite changes in HSCC2 group compared to WGMS data were also observed for four hyper-methylated GE-dMHBs and two hypo-methylated GE-dMHBs (Fig. 7E, Supplementary Fig. S10 in Additional file 2). This revealed the stability of DNA methylation changes of these GE-dMHBs observed between *S. aureus*-positive cows and healthy cows. This also suggests that the higher SCC and methylation alterations of cows in HSCC1 group may be due to *S. aureus* pathogens or pathogens causing mastitis. Then, we compared cows of HSCC1 and HSCC2 group with LSCC group, respectively to investigate the correlation between GE-dMHBs and mammary gland health condition. The methylation levels of all five hyper-methylated GE-dMHBs showed significant positive correlations with the SCC and milk somatic cell score (SCS) in HSCC1 and LSCC cows (FDR < 0.05 and *r* > 0.3), and significant negative correlation in HSCC2 and LSCC cows (FDR < 0.05 and *r* < − 0.3) (Supplementary Table S17F in Additional file 1). In contrast, the methylation levels of two hypo-methylated GE-dMHBs were negatively correlated with SCC and SCS in HSCC1 and LSCC cows, and positively correlated with SCC and SCS in HSCC2 and LSCC cows. Moreover, the correlation of GE-dMHBs with SCS (|*r*|: 0.56–0.71) was more significant and stronger than that with SCC (|*r*|: 0.33–0.59) (Supplementary Table S17F in Additional file 1). This observation suggests the potential of GE-dMHBs for capturing mammary gland health status and mastitis susceptibility in dairy cows. In addition, we also compared the methylation status of these seven GE-dMHBs by grouping cows according to their milk production traits, including test day milk yield (MY), milk protein, and fat percentages. The hyper- and hypo-methylated GE-dMHBs showed significantly higher and lower methylation levels in cows with high MY (> 40 kg/day), respectively, compared to cows with low MY (< 30 kg/day) (FDR < 0.05) (Fig. 7F, G, Supplementary Table S17F in Additional file 1 and Fig. S11 in Additional file 2). The methylation levels of the GE-dMHBs in cows with medium MY (20–30 kg/day) were similar with that in cows with high MY, but their differences compared with cows with low MY were less significant (0.05 < *p* value < 0.1). The difference in methylation levels of GE-dMHBs between cows with different MY suggests the possible association between DNA methylation alterations and milk production performance of dairy cows. However, no significant differences in the methylation levels of these GE-dMHBs were found between cows producing difference milk fat or protein percentages.

## Discussion

To date, few studies have addressed the potential influence of DNA methylation in the regulation of bovine mastitis caused by two prevalent pathogens, *E. coli* and *S. aureus* [26–28, 32, 33, 35], and in particular subclinical mastitis caused by *S. aureus* [36]. A larger proportion of them used in vitro infection strategies to induce clinical inflammatory responses followed by evaluation of the DNA methylation alterations related to mastitis [29, 35]. For instance, the strategy of using pathogen cell wall components to challenge bovine mammary epithelial cell lines has been commonly used [23–25]. Our study investigated the involvement of DNA methylation in relation to naturally occurring *S. aureus* subclinical mastitis. Milk somatic cells are capable to capture the global mammary gland responses to mastitis [58, 59]. This biological tissue is easy to collect without causing extra trauma to lactating cows and is thus considered as an efficient tissue for studying the related genetic and epigenetic changes and for future on farm testing applications. Yet, the DNA methylation characteristics of bovine milk somatic cells and their possible association with mastitis are still vague. In this regard, our study provides the first catalog of whole-genome DNA methylation alterations in milk somatic cells related to *S. aureus* subclinical mastitis.

Our data revealed abundant DNA methylation alterations during *S. aureus* subclinical mastitis at different layers including global DNA methylation levels at whole genome and per chromosome scales, general methylation level of genomic regulatory regions, REs, MHBs, and single cytosine sites. Globally, DNA methylation level was elevated in SAP group, mainly reflected in the higher global methylation level and hyper-methylation of about 80% of DMCs and dMHBs, compared to the control group. Consistent with our finding, the higher total DNA methylation level has also been found in Chinese Holstein cows with *S. aureus* mastitis [36] and in a mouse model for *S. aureus* mastitis [29] compared to healthy controls. This observation contrasts the lower global DNA methylation level observed in cows with mastitis caused by *E. coli* [34] and *S. uberis* [60] (which are pathogens belonging to environmental bacteria), and mammary epithelial cells in vitro challenged with bacterial lipopolysaccharide [23]. The changes of DNA methylation in different directions between mastitis caused by *S. aureus* and other pathogens indicate the potential of using DNA methylation information to detect subtle differences among bovine mastitis infections due to various causes. This may give a possible explanation to the division of 100 cows of HSCC group into two subgroups showing significant methylation changes in opposite directions in this study. Accordingly, we speculate that intermammary inflammation of cows in the HSCC1 subgroup with mean SCC (663,903 cells/mL) and that showed similar DNA methylation alterations with WGMS results in this study might have been caused by *S. aureus*. It is important to note, however, that the stage or duration of infection, such as the early (acute), middle (chronic), or last (just resolved infection) stages, could also contribute to the differentiation in DNA methylation status during subclinical mastitis. These aspects warrant further investigation to comprehensively understand the dynamics of DNA methylation changes in response to different stages of infection.

We also explored the DNA methylation alterations in genomic regulatory regions. Repeat elements, in particular the two abundant families (LINE-1 and tRNA-derived SINE), harbored abundant DNA methylation alterations related to *S. aureus* subclinical mastitis. The abundant accumulation of differential DNA methylation alterations in REs is consistent with the previously reported interactions between DNA methylation and RE activities, such as transposition, repression, or expression [61]. Although our understanding of the functions and regulatory mechanisms of REs is in its infancy, DNA methylation has already been found as an integral feature of RE control in mammals [62, 63]. The methylation of REs contributed to genome expansion and also provided opportunities for transcriptional control by RE-based regulatory sites [61, 64]. For instance, dynamic DNA methylation has been reported to reactivate REs as cryptic promoters that drove the widespread expression of oncogene expression across cancers [65]. In this study, we found two cases of correlation between RE, TSS, and DEGs (L1_Art-*PADI4* and CHRL-*CCAR1*) that are consistent with this hypothesis*.* It is worth noting that L1_Art and CHRL harbored the TSS of *PADI4* and *CCAR1*, respectively. Additionally, *PADI4* has been found as a putative candidate marker for susceptibility to subclinical mastitis in Norwegian Red cattle [66]. The strong positive correlation between hyper-methylation of L1_Art and CHRL and the upregulated expression of *PADI4* and *CCAR1* in this study revealed the possibility that aberrant DNA methylation may mediate activities of REs and thereby affect the transcriptional activities of corresponding genes during *S. aureus* subclinical mastitis. We need more functional validations to support this hypothesis; however, our current results suggest that exploration of DNA methylation alterations in REs may present novel avenues to further elucidate the regulatory mechanisms underlying bovine mastitis and even other diseases.

Comparing the DNA methylation level of single cytosine (DMC) or regions (DMR) (with fixed window size) has been the main strategy to evaluate DNA methylation alterations [67, 68]. However, there are limitations associated with these strategies. For example, the possible technical noise of measuring methylation levels of single cytosine sites may lead to false identification of DMCs. Although the approach of DMR could help to reduce this potential bias, it still has the limitation of subjectively choosing the window size and sliding step. Furthermore, the DNA methylation alterations in response to external stimulus, especially disease-causing pathogens, are mediated by corresponding changes in the activities of implicated enzymes [69]. The enzyme activities usually show coordinated regional effects leading to methylation haplotypes harboring adjacent CpG sites with similar methylation status [48]. Therefore, in our case, we employed the method of MHB that considers both the methylation levels of single CpG sites and the co-methylation between adjacent CpG sites, to investigate the DNA methylation alterations related to *S. aureus* subclinical mastitis. We identified numerous dMHBs with median length of 53 bp, which is much shorter than the window size (1000 bp) normally used for DMRs. More importantly, as shown in Fig. 4E, the methylation alterations of CpG sites, which were mostly identified as DMCs in the same dMHB, were tightly linked to each other. These relatively short dMHBs with coordinated methylation alterations exhibited significant differences between SAP and HC samples. Moreover, dominant dMHBs are the shorter fragment of DMRs enriched in DMCs. In this regard, the strategy of dMHB could help to overcome the limitations of DMC (high possibility of technical noise) and DMR (specifying window size). Hence, we suggest that identification of dMHBs could be a more reliable strategy to investigate the DNA methylation alterations related to *S. aureus* subclinical mastitis and other health and production traits.

We next explored the relationship between DNA methylation and gene expression, which is usually complex. DNA methylation patterns demonstrate cell-type specificity and are established by site-specific remodeling at regulatory regions during the dynamic mammalian differentiation events [70]. We observed the classic valley-like distribution of CpG methylation around TSS (Fig. 1), confirming the absence of DNA methylation at major gene regulatory sequences, especially promoter, as an important signature/guarantee for normal gene expression [71, 72]. In general, negative correlation has been found between methylation level of CpG sites located in gene promoters, first exons, and introns, and gene expression in the genome of various species, such as model pufferfish and frog, and different human tissues [45, 46, 73]. In line with this, our data revealed weak negative correlations between methylation levels of regulatory regions (promoters, first exons, and first introns) and the expression levels of corresponding genes at a whole genome scale. We also found that the changes in the methylation of these regulatory regions were also negatively correlated with the gene expression changes related to *S. aureus* subclinical mastitis. In addition, the major GE-dMHBs located in these regulatory regions also showed negative correlations with their overlapped DEGs. Our findings are consistent with previous reports that indicated the repressive effects of DNA methylation at regulatory regions on gene expression [70, 73, 74]. The TF motif identification in GE-dMHBs revealed the enrichment of some common factors like IRF2, SP4, EGR1, and KLF4 that have been found to regulate gene expression through binding to CpG-rich promoters [51, 52]. This further supports the involvement of DNA methylation alterations at regulatory regions, particularly GE-dMHBs, in the regulation of gene expression in response to *S. aureus* subclinical mastitis.

Our results also revealed that genes harboring DNA methylation alterations may have immune-related functions during *S. aureus* subclinical mastitis. For instance, *Staphylococcus aureus* infection (bta05150) pathway was significantly enriched by genes (e.g., *CATHL2*, *CATHL3*, *KRT18*, *FGG*) harboring DMCs at their first exons as well as differential genes (identified by MethGET) having significant DNA methylation changes in their regulatory regions. For example, *KRT18* and *FGG* are key mediators that bind with microbial surface components recognizing matrix molecules of pathogens like *S. aureus* and thereby indirectly affect their colonialization [75]. Our data revealed the downregulation of these two genes and the higher general methylation levels at their regulatory regions. Moreover, the upregulated *C5AR1*, *FCGR3A*, and *FCAR* that have indirect effects on inhibition of chemotaxis and phagocyte activation [76], all had lower general methylation levels at their regulatory regions. We also found low methylation level of regulatory region of upregulated *CATHL3*, which may affect its antimicrobial activity. We therefore speculate that the abnormal expression of these genes may be associated with the inverse changes of the methylation states of their regulatory regions, which potentially contribute to the regulation of mammary gland immune defense against *S. aureus*. In support of this, our results of the functional enrichment of GE-dMHBs (correlated with their overlapped DEGs) further suggest the involvement of DNA methylation alterations in the regulation of immune functions. Upon *S. aureus* invasion of the mammary gland, a series of immune responses are triggered, such as recruitment of leukocytes [77] and production of cytokines [78]. Consistently, the upregulated DEGs that negatively correlated with their overlapped hypo-methylated GE-dMHBs were enriched in multiple KEGG pathways with key immune functions, such as chemokine signaling, cytokine-cytokine receptor interaction, and activities of immune-related cells (leukocyte, Th17, natural killer cell and B cell, etc.). This suggests that the hypo-methylation of GE-dMHBs at regulatory regions may play roles in the upregulated expression of these DEGs, which probably contributed to increase the bacteriostatic and bactericidal activities required to kill *S. aureus* [77, 79]. On the other hand, *S. aureus* invasion of mammary gland cells during long-term subclinical mastitis cause damage to the epithelial cell lining of the mammary gland, which impairs its recovery ability and affects milk production [80]. We found that downregulated DEGs that were negatively correlated with their overlapped hyper-methylated GE-dMHBs were enriched in some important biological functions, such as cellular activities related to cell migration and localization, cell development, especially epithelial cell differentiation and epithelium development, and metabolic processes. The hyper-methylation of related GE-dMHBs might have played a role in the downregulated expression of these DEGs and thus reflects a possible regulatory mechanism underlying impaired cellular activities and decreased productivity of the mammary gland during subclinical mastitis. In this regard, we believe that DNA methylations, especially GE-dMHBs, could be a possible mechanism of the genetic regulation of mammary gland responses during *S. aureus* subclinical mastitis. However, our current findings could not further pin-point the causation between DNA methylation alterations and gene expression changes during *S. aureus* subclinical mastitis.

A plethora of studies in the subject area of epigenetics have revealed that DNA methylation alterations could provide new avenues to investigate the portion (“black box”) of phenotypic variations unexplained by genetic variation [17]. Epigenetic information is valuable as it further understanding of how environmental influences on the genome shape phenotypic expression of livestock production traits, which may contribute to the improvement of breeding and farm management strategies [16, 19, 81–83]. Therefore, by integrating GE-dMHBs and their significantly correlated DMGs, we identified discriminant signatures that differentiated between SAP and HC groups and which have the potential to be further validated as candidate markers for *S. aureus* subclinical mastitis. It is worth noting that the pro-GE-dMHB (Chr29:46226206:46226215) located in the promoter of *CPT1A* showed the highest potential to drive the epigenetic variations between SAP and HC group. *CPT1A* is a key gene related to fatty acid oxidation and play important roles on lipid metabolism in dairy cattle [84]. A previous study identified upregulated expression of *CPT1A* in response to intra-mammary lipopolysaccharide challenge [85], which agrees with its upregulation in milk somatic cells during *S. aureus* subclinical mastitis in this study. The strong negative correlation between this pro-GE-dMHB and the expression of *CPT1A* further strengthens the association of this epigenetic signature with *S. aureus* subclinical mastitis. The hypo-methylation status of this pro-GE-dMHB may also be an epigenetic regulator of the increased expression of *CPT1A* in response to *S. aureus* infection. Unfortunately, we did not find appropriate primers for this pro-GE-dMHB to validate its methylation status in a larger sample size. However, the validation of other dMHBs revealed the stability of methylation alterations of dMHBs between cows with high and low SCC. Therefore, we believe that this pro-GE-dMHB has great potential to be a candidate epigenetic maker for *S. aureus* subclinical mastitis, but more functional validations are needed. In addition, GE-dMHBs offer notable advantages inherent to dMHBs. Their relatively short length and high density of co-methylated cytosine sites make them particularly amenable to analysis, ensuring higher accuracy and reliability with currently available technologies. This intrinsic characteristic enhances their potential for in-depth exploration and interpretation. Looking ahead, the ongoing advancements in technology, particularly the potential development of specialized DNA methylation assays or chips tailored for bovine genomes, offer the prospect for cost-effective and high-throughput detection of GE-dMHBs in large sample sizes. This technological stride not only presents an opportunity for widespread identification but also holds the potential to validate further the candidacy of our identified discriminant GE-dMHBs as promising epigenetic biomarkers for subclinical mastitis. This progress will not only facilitates the widespread identification of these epigenetic features but also paves the way for their practical application in the field. The applications could span various areas in dairy farming such as incorporation in to current genomic breeding strategies, evaluation of calf or heifer production performance, early-stage diagnosis of subclinical mastitis, and development for therapeutic applications. The versatility of GE-dMHBs, coupled with evolving technological capabilities, position them as valuable tools for advancing precision management practices in the dairy industry.

In this study, we profiled the DNA methylation pattern of milk somatic cells and investigated the alterations related to *S. aureus* subclinical mastitis. Milk somatic cells are a mixture of multiple types of cells, each of which may have different epigenetic responses to *S. aureus* infection due to the high cell-type specificity of DNA methylation. This may be considered a limitation in this study since the detected DNA methylation alterations are possibly coming from these various cell types probably caused by the changes in milk somatic cell composition during mastitis, especially a higher proportion of immune cell populations responding to *S. aureus* presence in SAP samples [58, 59]. The cell-type specificity of DNA methylation alterations nor the contribution due to the changed proportion of different cell types in SAP group could not be tested by the WGMS technology used in this study. Therefore, future studies would benefit from having access to robust technologies like single-cell sequencing or spatial profiling of mammary gland tissues, which will help to better investigate the spatiotemporal specific DNA methylation alterations of the different cell types during *S. aureus* subclinical mastitis. However, since the milk somatic cells captures most cell populations involved in the mammary response to infection and are easy to collect, our study has demonstrated the presence of DNA methylation alterations of relevance to *S. aureus* subclinical mastitis that can be further validated/developed for practical on-farm applications. Besides, we demonstrated the significant correlation between DNA methylation alterations and gene expression changes related to *S. aureus* subclinical mastitis. But we could not determine whether these identified DNA methylation alterations were the cause or the consequence of the gene expression changes with the current data. It is crucial to acknowledge the intricate relationship between these two molecular processes. Transcript activity changes can induce alterations in DNA methylation patterns, and conversely changes in methylation patterns can impact gene expression. This dynamic interaction forms a complex feedback loop, where each component has the potential to influence the other. The precise interplay between DNA methylation and gene expression in the context of *S. aureus* subclinical mastitis is a captivating avenue for further exploration in order to offer further insights into the nuanced regulatory mechanisms at play. Another limitation of this study is that the phenotype of cows used for population analysis was based on their SCC records without bacteriological examination to give details on the infecting pathogens or specific health status. Therefore, we could not further check the possible reasons for the obvious clustering of cows with HSCC into two sub-groups with different DNA methylation alterations of dMHBs in this study. Further investigations with detailed mammary gland health condition will be needed to validate the potential of DNA methylation alterations to detect subtle differences among bovine mastitis due to various causes. Finally, as is the case for DNA methylation studies in human diseases, the analysis of DNA methylation changes will further understanding of the underlying mechanisms of *S. aureus* subclinical mastitis and provide new avenues for improving mastitis control strategies.

## Conclusions

In this study, we comprehensively analyzed the whole-genome DNA methylation pattern of milk somatic cells and identified a wide variety of DNA methylation alterations related to *S. aureus* subclinical mastitis. Firstly, the global DNA methylation level trend was higher during *S. aureus* subclinical mastitis. Secondly, 3.36 million DMCs and 153,783 dMHBs (which considers the co-methylation status of adjacent CpG sites) were identified. Thirdly, we found that DNA methylation alterations in regulatory regions displayed inverse relationships with gene expression which suggest roles in the regulation of gene expression during *S. aureus* mastitis. Out of 6435 GE-dMHBs at the regulatory regions of DEGs with significant correlation with their overlapped DEGs, nine GE-dMHBs emerged with discriminant signatures that may drive the epigenetic changes between SAP and HC groups. Additionally, the DNA methylation alterations are involved in the regulation of immune responses to *S. aureus* subclinical mastitis as revealed by the significant enrichment of genes harboring DNA methylation alterations in immune- and disease-related pathways. Finally, the stability of DNA methylation alterations of seven dMHBs and their association with mammary gland healthy was validated in a larger sample size. Overall, our findings provided a comprehensive DNA methylation profile of milk somatic cells and abundant DNA methylation alterations relevant to *S. aureus* subclinical mastitis, and revealed the possible roles of DNA methylation in the regulation of health and milk production performance of dairy cows. Finally, our results may contribute to promote the application of epigenetics information in furthering understanding of host responses to mastitis pathogens and in improving mastitis control strategies in the dairy industry.

## Methods

### Cow selection and sample collection

Lactating Canadian Holstein cows with naturally occurring *S. aureus* subclinical mastitis were selected from five commercial farms in Quebec. The details about cow selection and sample collection are described in our previous transcriptome study [38]. Briefly, we firstly selected cows with consecutively high (> 350,000 cells/mL) (HSCC) or low (< 150,000 cells/mL) (LSCC) SCC over a period of 3 months or more. Milk samples (~ 5 mL) from each mammary quarter of cows in HSCC group or a composite sample from each cow in the LSCC group were aseptically collected and subjected to bacteriological examination (Biovet laboratories, St-Hyacinthe, QC, Canada). Next, fifteen cows of LSCC group negative to all mastitis pathogens tested and eighteen cows of HSCC with one or more quarters positive to *S. aureus* only, were enrolled as healthy control (HC) and *S. aureus* positive (SAP) groups, respectively. About 200 mL of composite milk from each HC cow and 200 mL of milk from one positive quarter of each SAP cow was collected as the final milk samples. Due to the short period (3–5 days) between receiving bacteriological examination and final milk sampling, a small portion (~ 2 mL) of final milk samples per cow of both SAP and HC group was sent for bacteriological examination as validation of initial bacteriological results. A total of sixteen SAP quarters (cows) and ten HC cows with consistent bacteriological results were finally kept for subsequent analyses (Supplementary Table S1 in Additional file 1). The final milk samples were placed on ice and immediately transported to our laboratory for isolation of milk somatic cells by centrifugation at low speed (1500 × *g*, 15 min, 4 ℃) with washing twice (PBS added and centrifugation at 1500 × *g* for 15 min at 4 ℃). The isolated milk somatic cells were stored at − 20 °C for DNA isolation.

### DNA isolation and WGMS

DNeasy Blood and Tissue Kit (Qiagen Inc., Toronto, ON, Canada) was used to isolate genomic DNA from milk somatic cells, and the quality and quantity was checked with Quant-iT™ PicoGreen® dsDNA Assay Kit (Life Technologies, Burlington, ON, Canada). The bisulfite treatment commonly employed in whole genome bisulfite sequencing can induce damages such as fragmentation, DNA loss, and biased sequencing data. To mitigate these effects, this study utilized the NEBNext® Enzymatic Methyl-seq kit (New England BioLabs Ltd., Whitby, ON, Canada) for WGMS library preparation. This kit employs enzymatic reactions, contrasting with bisulfite conversion, to detect cytosine methylation (5mC) [86]. The two-step enzymatic process involves the initial conversion of 5mC into products resistant to APOBEC3A deamination by TET2. Subsequently, APOBEC3A catalyzes the conversion of unmodified (unmethylated) cytosine to uracil. This approach offers an alternative to traditional bisulfite treatment, enhancing the robustness and accuracy of methylation detection. Next, the quality of WGMS libraries were checked by using Kapa Illumina GA with Revised Primers-SYBR Fast Universal kit (Kapa Biosystems Inc., Wilmington, MA, US), and the average size of fragments was determined with a LabChip GX (PerkinElmer Inc., Waltham, MA, US) instrument. Then, the libraries were normalized and equimolar pooled for denaturation in 0.05 N NaOH and neutralization in HT1 buffer. The library pool was then loaded at 225 pM on an Illumina NovaSeq S4 lane using Xp protocol according to the manufacturer’s recommendations. The sequencing was run for 2 × 100 cycles in paired-end mode that a phiX library was mixed with library at 1% level as control. Program RTA (version 3.4.4) and bcl2fastq2 (version 2.20) were used for base calling and generating fastq reads, respectively.

### Raw data processing and differential methylation site identification

The raw WGMS data was processed using the standard pipeline for DNA methylation sequencing (methylseq from nf-core) [87] with modifications. Briefly, the first 8 bp of each read was removed by selecting “EM Seq” trimming profile to avoid potential bias towards non-methylation at the end of reads caused by end repairing. FastQC (version 0.11.9) and Trim Galore! (Version 0.6.6) were used for generating sequence quality report and trimming (adapter sequences and low-quality reads), respectively. The clean reads with high quality were then merged and aligned to the bovine reference genome (ARS-UCD1.2) using bowtie2 under Bismark (version 0.22.0) [88]. The BAM files was generated using Samtools (version 1.11) [89]. Identification of methylation sites in the context of CpG, CHG, and CHH (H means A or T or C) was performed with bismark_methylation_extractor under Bismark. Methylation sites detected in ≥ 80% of samples per group with ≥ 7 reads coverage depth were retained for the next-step analyses.

DNA methylation levels of detected cytosines were compared between SAP and HC groups using Methylkit (version 3.12) [47] to identify DMCs in three contexts (CpG, CHG, and CHH). Possible effect factors, including farm, lactation stage, and parity, were included to eliminate batch effects and decrease random noise. DMC was defined as methylation site that passed two filter thresholds, including > 20% difference in methylation level and *q*-value < 0.05. In addition, the methylation level comparisons between SAP and HC groups were processed by two-tailed *t* test at the scale of the whole genome, chromosomes, and genomic regions (such as gene features and regions related to CGI). The *p*-value was adjusted by Benjamini and Hochberg false discovery rate (FDR) correction [90], and FDR < 0.05 was used to define significant difference between groups.

### Methylation level prediction and comparison for repeat elements

The methylation level was predicted for LINE-1 and tRNA-derived SINEs, which are the most abundant LINE and SINE superfamily in the bovine genome, respectively, by using the random forest-based algorithm (corresponding R package, REMP, version 1.22.0) [91]. In brief, the beta value of all qualified CpG sites and the annotation data of REs were used for the calculation of REMP. The annotation file of REs was downloaded from UCSC Table browser by choosing the track “ReapeatMasker”. Since nearby CpG sites tend to be co-methylated, REMP predict the methylation level of target CpG sites located in REs by using neighboring CpG sites within a flanking window size (1000 bp). To increase the reliability of prediction, target CpG sites with at least two neighboring CpG sites within the flanking window were kept for the prediction. Then the random forest model was used to predict the methylation level of target CpG sites located in LINE-1 elements and tRNA-derived SINEs per sample by using function “remp()” with default parameters. REs with at least two predicted CpG sites were kept, whose methylation levels were further calculated by averaging the predicted methylation of CpG sites in them. Then the genomic region indicators, including protein-coding genes, TSS, 5′UTR, coding DNA sequence regions, exon, and 3′UTR, were added for LINE-1 elements and tRNA-derived SINEs. The methylation level of LINE-1 elements and tRNA-derived SINEs were then compared by two-tailed *t* test between SAP and HC group, and *p*-value was adjusted by Benjamini and Hochberg false discovery rate (FDR) correction [90]. FDR < 0.05 was used to define the significance of differences between two groups. Besides, the methylation level of LINE-1 elements and tRNA-derived SINEs were correlated to the healthy status of cows (SAP or HC) by using Point-Biserial Correlation Coefficient (r_pb_), and significant correlation was defined by | r_pb_ |> 0.3 and adjusted *p*-value (FDR) < 0.05.

### Methylation haplotype blocks identification and comparison

To investigate the regions with highly coordinated methylation, MONOD2 was used to identify MHBs [48]. Briefly, the clean and qualified reads of all samples were pooled to split the reference genome (ARS-UCD1.2) into non-overlapping “sequenceable and mappable” segments. The methylation haplotypes were identified from mapped reads inside each segment. Methylation linkage disequilibrium was then calculated for methylation haplotypes of all samples to identify MHBs, which was defined as the regions harboring ≥ 3 CpG sites and having ≥ 0.5 *r*^2^ value for any two adjacent CpG sites. Next, the normalized methylation level (methylated haplotype load, MHL) was calculated for each MHB per sample. Two-tailed Student’s *t* test was used to compare the methylation status of MHBs between SAP and HC groups to identify dMHBs. The *p*-value was adjusted as FDR, and dMHB was defined as MHB having ≥ 20% difference in MHL between groups and FDR < 0.05.

### Annotation and enrichment analysis

The genome structure annotation file of the bovine reference genome (ARS-UCD1.2), including position information of genes, REs and CGI was downloaded from the UCSC Table browser. The 2000-bp region upstream of TSS was considered as the promoter, while “downstream region” was referred to as the 2000-bp region downstream of the transcription termination site. Relative to CGI, CGI shores, CGI shelves, and CGI desserts were defined as 0 ~ 2000 bp, 2001 ~ 4000 bp, and > 4000 bp regions upstream/downstream of CGI, respectively. Reported QTLs for mastitis and immune capacity were download from CattleQTLdb (https://www.animalgenome.org/cgi-bin/QTLdb/BT/index, accessed at 20^th^ January, 2023). The REs were obtained from the track of “RepeatMasker”. The annotatr (version 3.12) was used to check the overlapping of methylation sites, DMCs, MHBs, and dMHBs with these genomic regions. In addition, regioneR (version 1.28.0) was used for the enrichment analysis of dMHBs in genomic regions that all identified MHBs were set as the background (permutation test: 1000) [92].

### Correlation between DNA methylation and transcriptome

MethGET program [43] was utilized to investigate the correlation between DNA methylation and gene expression. In brief, the three input files used for MethGET analysis included the DNA methylation CGmap file (converted from methylation coverage report files generated by Bismark), normalized gene expression file of the same samples [38], and gene annotation GTF file (converted from GCF_002263795.1). First of all, the general methylation levels of the different genomic functional regions, including promoter, first exon, first intron, exons, introns, and gene body, were calculated by averaging the methylation levels of all qualified CpG sites in the corresponding regions. Then, Pearson correlation coefficient was used to investigate the correlation between DNA methylation levels of the different regions and gene expression levels per sample at a scope of the whole genome. Besides, the changes in general DNA methylation levels of the different genomic functional regions and gene expression changes between SAP and HC groups were also correlated by Pearson correlation coefficient on a genome-wide scale. Then, *p*-value < 5 × 10^−8^ was used as the threshold of significant genome-wide correlation. Furthermore, the Gaussian Mixture Model from the machine learning package (scikit-learn) in python was used to identify genes with significant changes of general DNA methylation level of specific regulatory regions (promoter, first exon or first intron) and/or gene expression levels (referred to as differential genes) [93]. The *p*-value calculated by Gaussian Mixture Model less than 0.005 was used to define significant differential genes. The identified differential genes were further filtered by only keeping those having more than 10% difference in general methylation changes of corresponding regulatory regions and greater than 1 of absolute log_2_FC in gene expression changes.

In addition, Spearman’s rank correlation coefficient (rho) was used to correlate the methylation status of dMHBs that overlapped with promoter, first exon or first intron of DEGs, and the expression level of their overlapped DEGs. *P*-value was adjusted by FDR through independent hypothesis weighting (IHW) framework [94]. Significant correlation between the methylation status of dMHBs and the expression level of the corresponding overlapped genes was defined as |rho|> 0.3 and FDR < 0.05.

### Identification of candidate discriminant signatures

The DIABLO framework (Data Integration Analysis for Biomarker discovery using a Latent cOmponents) from mixOmics (version 6.22.0) [95] was used to identify highly correlated GE-dMHBs (pro-GE-dMHBs) and DEGs as candidate signatures that could explain the categorical outcome between SAP and HC groups. The core DIABLO method is designed to integrate multiple heterogeneous datasets from same biological samples based on a variant of the multivariate methodology Generalized Canonical Correlation Analysis (GCCA) [95, 96]. The DNA methylation status of GE-dMHBs (pro-GE-dMHBs) and the expression level of corresponding overlapped DEGs were used as two input datasets for DIABLO. The full weighted design was used to get the trade-off between maximizing correlation between input datasets and the discrimination of selected signatures. The two key parameters, including number of components and number of signatures, were set following the authors’ recommendation [95]. According to the recommend K-1 components (K: number of groups) to get the best classification performance, the number of components was set as one in this study. The number of signatures with best predictive performance were determined by performing repeated cross-validation (5-fold cross-validation, 50 times repeat) thorough “tune” function.

### Target bisulfite amplicon sequencing for validation of GE-dMHBs in a larger sample size

The methylation levels of seven GE-dMHBs were further checked in a bigger sample size by next-generation sequencing-based TBAS method. A total of 200 cows were sampled from nine commercial farms in Quebec, Canada, and grouped according to their SCC records as high SCC (HSCC, ≥ 200,000 cell/mL, *n* = 100) and low SCC (LSCC, ≤ 100,000 cell/mL, *n* = 100). Milk samples were collected from the cows on the same day of milk collection for monthly DHI test. Milk somatic cell isolation, DNA isolation and qualification were done as described above. Then the qualified DNA samples were used for TBAS. Briefly, candidate discriminant GE-dMHBs (*n* = 5) and further top GE-dMHBS (*n* = 9) were selected for primer design using the online MethPrimer software [97] for TBAS application (Supplementary Table S17 in Additional file 1). Unfortunately, the genomic regions of the 5 candidate discriminant GE-dMHBs and 2 top GE dMHBs did not support the design of appropriate primers. One microgram of genomic DNA was bisulfite converted using the ZYMO EZ DNA Methylation-Gold Kit (Zymo Research, Irvine, CA, USA). Then, one twentieth of the eluted DNA conversion products were used as template for PCR amplification with 35 cycles using KAPA HiFi HotStart Uracil + ReadyMix PCR Kit (Kapa Biosystems, Wilmington, MA, USA). The PCR products of each GE-dMHBs were then pooled equimolarly as the sequencing library for each sample, 5’-phosphorylated, 3’-dA-tailed and ligated to barcoded adapter by using T4 DNA ligase (New England Biolabs, Ipswich, MA, USA). Barcoded libraries of all samples were sequenced on the Illumina Hiseq platform using paired-end (150 bp) strategy.

The raw data of target bisulfite sequencing was firstly filtered to remove adapter sequences and low-quality sequences (having greater than 5% unknown bases or having greater than 50% of bases with Phred score less than 20) using Trimmomatic (version 0.36) [98]. Then the clean sequencing data was aligned to the bovine reference genome (ARS-UCD1.2) using BSMAP (version 2.73) [99]. The beta methylation level was calculated for each detected CpG site. The correlation between CpG sites located in the same GE-dMHB was evaluated by using the R package EnMCB (version 1.10.0) [100]. The significance of difference in methylation level of each CpG sites and GE-dMHBs were compared between groups by using two-tailed *t* test. The *p* value was adjusted as FDR, that FDR < 0.05 was used to define significant difference. And then another R package CpGassoc (version 2.60) [101] was used to calculate the association between methylation level of GE-dMHBs and SCC as well as milk production traits (milk yield, milk fat, and protein percentages) (Supplementary Table S17 in Additional file 1).

### Functional annotation analysis

The gene functional enrichment analysis, including GO and KEGG pathway, was performed with ClusterProfiler (Version 4.2.2) [54] for selected gene lists, which represent association with GO terms and KEGG pathways without indicating the direction of regulation. The method “simplify” was used to reduce redundancy of enriched GO terms caused by the parent-child structure of GO terms. Significant enrichment of GO terms or KEGG pathways were considered as having adjusted *p*-value (FDR) < 0.05. The enrichment results were visualized with enrichplot (version 1.16.1). Function “AME” and “runDreme” from MEMEs (version 1.6.0) [53] were used to detect the transcription factor motif enrichment and de novo discovery of motifs in GE-dMHBs, respectively. Known motifs (width 6 to 35 base pairs) in mammals from JASPR^2022^ core vertebrates database (https://jaspar.elixir.no/search?q=&collection=CORE&tax_group=vertebrates) corresponding to expressed genes in milk somatic cells in this study were set as the relevant candidate motif database to improve statistical power of motif search by MEMEs.

### Supplementary Information

**Additional file 1: Supplementary Table S1.** Detail information on cows selected for whole genome DNA methylation sequencing. **Supplementary Table S2.** General statistics on whole genome DNA methylation sequencing data. **Supplementary Table S3.** Comparisons of methylation level between cows with *S. aureus *subclinical mastitis and healthy control cows. **Supplementary Table S4.** List of differentially methylated cytosines in the context of CpG. **Supplementary Table S5.** List of differentially methylated cytosines in the context of CHG and CHH. **Supplementary Table S6.** Distribution of differentially methylated cytosines among chromosomes and genetic features. **Supplementary Table S7.** Distribution of differentially methylated cytosines in genes. **Supplementary Table S8.** Functional enrichment of genes harboring differentially methylated cytosines at different genomic functional regions. **Supplementary Table S9.** Predicted methylation level for LINE-1 and tRNA-derived SINEs and comparisons between cows with *S. aureus *subclinical mastitis and healthy control cows. **Supplementary Table S10.** Differential methylation haplotype blocks. **Supplementary Table S11.** Differential genes with significant changes in general methylation level of regulatory regions and gene expression changes. **Supplementary Table S12.** Functional enrichment for differential genes with significant changes in general methylation level of regulatory regions and gene expression changes. **Supplementary Table S13.** Correlation between dMHBs located at regulatory regions and their overlapped differentially expressed genes. **Supplementary Table S14.** Motif enrichment and identification in GE-dMHBs. **Supplementary Table S15.** Functional enrichment for GE-dMHBs. **Supplementary Table S16.** Candidate discriminant signatures selected from GE-dMHBs and their overlapped genes. **Supplementary Table S17.** Information about samples and primers used for TBAS and results of selected GE-dMHBs.

**Additional file 2: Supplementary Figure S1.** Correlation between gene expression and methylation levels of different genetic regions at a scale of the whole genome. **Supplementary Figure S2.** Comparison of the global methylation levels of different genetic regions between SAP and HC group. **Supplementary Figure S3.** Comparison of the global methylation levels of CpG islands (CGI), shores and shelves between SAP and HC groups. **Supplementary Figure S4.** Comparison of global methylation level of cytosines in the context of CHG and CHH between SAP and HC group. **Supplementary Figure S5.** Comparison of global methylation level of cytosines in the context of CHG located in CpG islands (CGI), shores and shelves between SAP and HC groups. **Supplementary Figure S6.** Density of CpG sites in LINE-1 and t-RNA-derived SINEs. **Supplementary Figure S7.** Summary of identified methylation haplotype blocks (MHBs). **Supplementary Figure S8.** Identification of differential genes with significant changes in their gene expression level and the general methylation level of first exon (A) and first intron (B). **Supplementary Figure S9.** de novo identified motifs in GE-dMHBs. **Supplementary Figure S10.** Boxplots showing the methylation difference of select GE-dMHBs between cows with high or low milk somatic cell count (SCC). **Supplementary Figure S11.** Boxplots showing the methylation difference of selected GE-dMHBs between cows with high or low milk yield (MY).

## Data Availability

All data generated or analyzed during this study are included in this published article and sequence data and supplementary tables are placed in publicly available repositories. The whole genome DNA methylation sequencing data is available in the NCBI BioProject database (https://www.ncbi.nlm.nih.gov/bioproject/) under accession number PRJNA962142. The supplementary tables are available in Figshare repository at https://figshare.com/s/7d2df22152dd897e42a6 [102].

## Abbreviations

BP: Biological process
CC: Cellular component
CGI: CpG island
Chr: Chromosome
DEGs: Differentially expressed genes
DMCs: Differentially methylated cytosine sites
dMHBs: Differential methylation haplotype blocks
*E. coli*: *Escherichia coli*
FE-dMHBs: Differential methylation haplotype blocks overlapping with the first exon of genes
FE-GE-dMHBs: Gene expression correlated differential methylation haplotype blocks that overlapped with first exon of genes
FI-dMHBs: Differential methylation haplotype blocks overlapping with the first intron of genes
FI-GE-dMHBs: Gene expression correlated differential methylation haplotype blocks that overlapped with first intron of genes
GE-dMHBs: Gene expression correlated differential methylation haplotype blocks
GO: Gene ontology
HC: Healthy control
HSCC: High somatic cell count in milk
HSCC1: First subgroup of cows with high somatic cell count in milk
HSCC2: Second subgroup of cows with high somatic cell count in milk
KEGG: Kyoto Encyclopedia of Genes and Genomes
LINEs: Long interspersed nuclear elements
LSCC: Low somatic cell count in milk
LTR: Long terminal repeat
MF: Molecule function
MHBs: Methylation haplotype blocks
MHL: Methylation haplotype load
MY: Milk yield
pro-dMHBs: Differential methylation haplotype blocks overlapping with the promoter region of genes
pro-GE-dMHBs: Gene expression correlated differential methylation haplotype blocks that overlapped with promoter region of genes
REs: Repeat elements
*S. aureus*: *Staphylococcus aureus*
SAP: *S. aureus*-Positive
SCC: Somatic cell count
SINEs: Short interspersed nuclear elements
TSS: Transcriptional start sites
WGMS: Whole genome methylome sequencing
