# Supplementary material for: DNA methylation haplotype block signatures responding to Staphylococcus aureus subclinical mastitis and association with production and health traits
Source: BMC Biol. 2024 Mar 14;22:65. doi: 10.1186/s12915-024-01843-y (PMC10941392; doi:10.1186/s12915-024-01843-y)
Supplement: Supplementary file 2 — Additional file 2: Supplementary Figure S1. Correlation between gene expression and methylation levels of different genetic regions at a scale of the whole genome. Supplementary Figure S2. Comparison of the global methylation levels of different genetic regions between SAP and HC group. Supplementary Figure S3. Comparison of the global methylation levels of CpG islands (CGI), shores and shelves between SAP and HC groups. Supplementary Figure S4. Comparison of global methylation level of cytosines in the context of CHG and CHH between SAP and HC group. Supplementary Figure S5. Comparison of global methylation level of cytosines in the context of CHG located in CpG islands (CGI), shores and shelves between SAP and HC groups. Supplementary Figure S6. Density of CpG sites in LINE-1 and t-RNA-derived SINEs. Supplementary Figure S7. Summary of identified methylation haplotype blocks (MHBs). Supplementary Figure S8. Identification of differential genes with significant changes in their gene expression level and the general methylation level of first exon (A) and first intron (B). Supplementary Figure S9. de novo identified motifs in GE-dMHBs. Supplementary Figure S10. Boxplots showing the methylation difference of select GE-dMHBs between cows with high or low milk somatic cell count (SCC). Supplementary Figure S11. Boxplots showing the methylation difference of selected GE-dMHBs between cows with high or low milk yield (MY). [file 12915_2024_1843_MOESM2_ESM.pdf]

Content of supplemental figures

Supplemental Figure S1 Correlation between gene expression and methylation levels of different genetic regions at a scale of the whole genome. 1

Supplemental Figure S2 Comparison of the global methylation levels of different genetic regions between SAP and HC group. 2

Supplemental Figure S3 Comparison of the global methylation levels of CpG islands (CGI), shores and shelves between SAP and HC groups. 3

Supplemental Figure S4 Comparison of global methylation level of cytosines in the context of CHG and CHH between SAP and HC group. 4

Supplemental Figure S5 Comparison of global methylation level of cytosines in the context of CHG located in CpG islands (CGI), shores and shelves between SAP and HC groups. A 5

Supplemental Figure S6 Density of CpG sites in LINE-1 and t-RNA-derived SINEs. 6

Supplemental Figure S7 Summary of identified methylation haplotype blocks (MHBs). 7

Supplemental Figure S8 Identification of differential genes with significant changes in their gene expression level and the general methylation level of first exon (A) and first intron (B). 8

Supplemental Figure S9 *de novo* identified motifs in GE-dMHBs 9

Supplemental Figure S10 Boxplots showing the methylation difference of select GE-dMHBs between cows with high or low milk somatic cell count (SCC). 10

Supplemental Figure S11 Boxplots showing the methylation difference of selected GE-dMHBs between cows with high or low milk yield (MY). 11

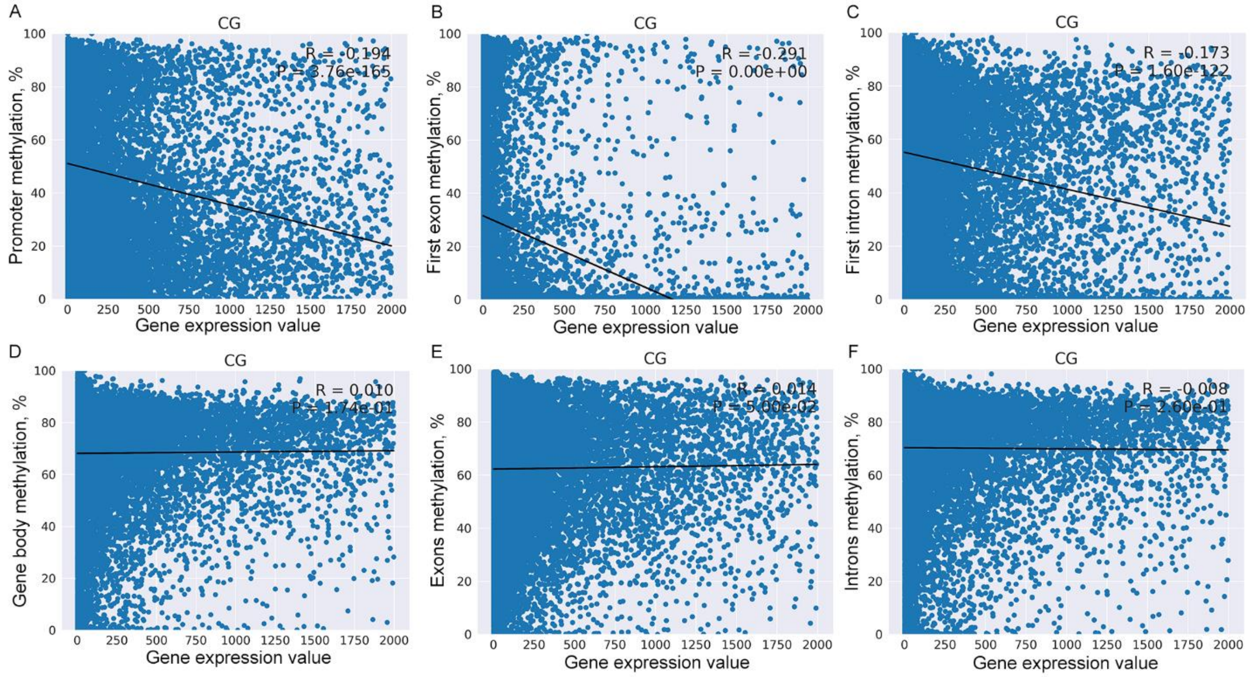

Supplemental Figure S1 Correlation between gene expression and methylation levels of different genetic regions at a scale of the whole genome. **A-F** represent the global methylation level of promoter, first exon, first intron, gene body which includes all exons and introns of a gene, exons and introns, respectively. Only qualified cytosines in the context of CpG (with at least seven reads coverage in ≥ 80% of samples per group) were used for calculation of the general methylation level of each region.

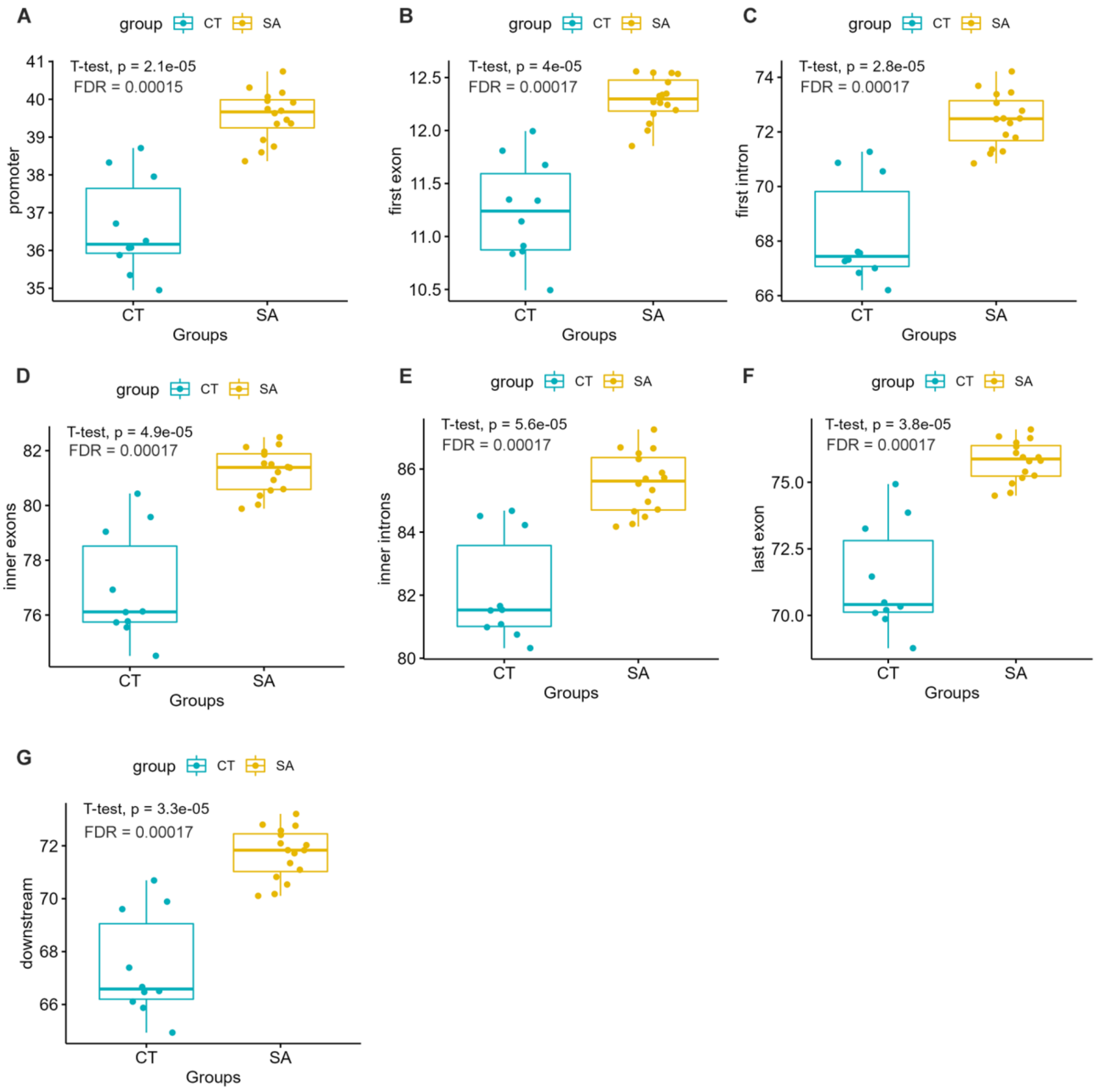

Supplemental Figure S2 Comparison of the global methylation levels of different genetic regions between SAP and HC group. **A-G** represent the global methylation levels of promoter, first exon, first intron, inner exons, inner introns, last exon, and downstream region (2 kb downstream of transcript terminate site), respectively. Only qualified cytosines in the context of CpG were used for the calculation of the general methylation level of each region.

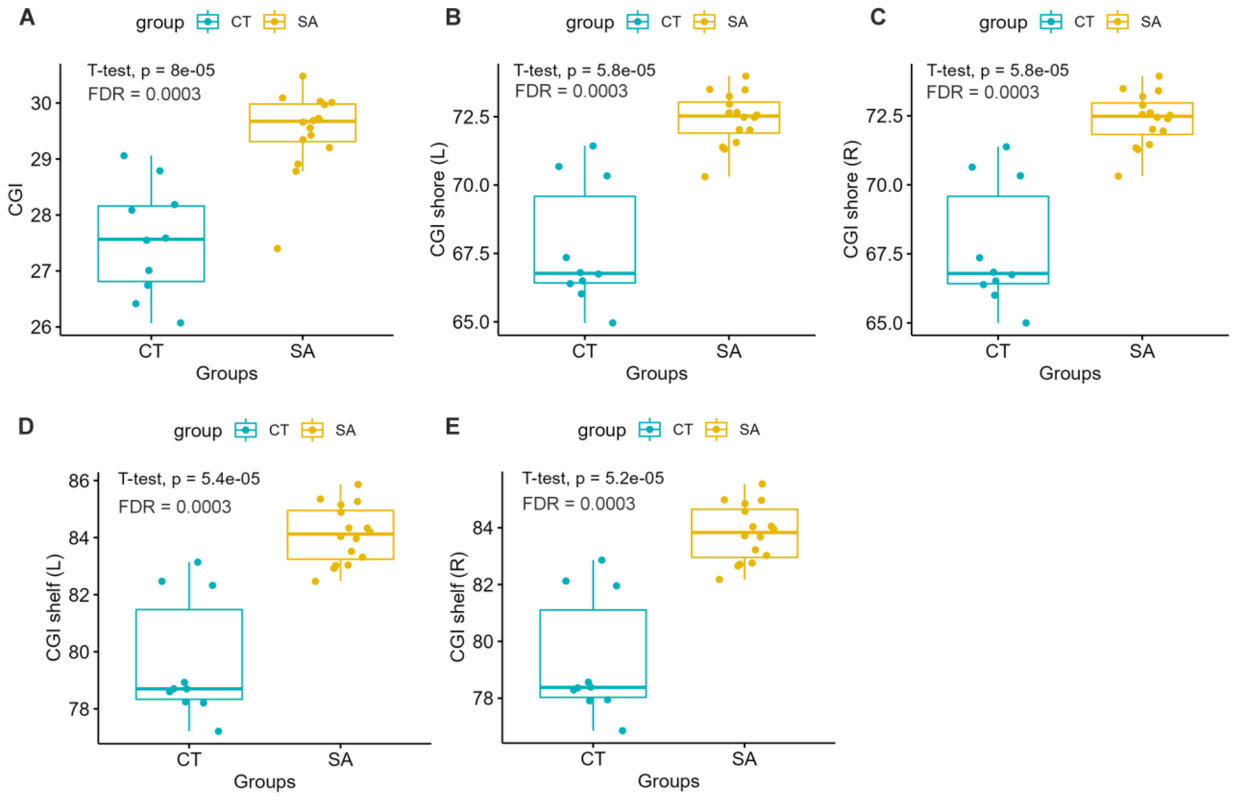

Supplemental Figure S3 Comparison of the global methylation levels of CpG islands (CGI), shores and shelves between SAP and HC groups. **A-E** represent the global methylation levels of CGI, left CGI shore, right CGI shore, left CGI shelf and right CGI shelf, respectively. Only qualified cytosines in the context of CpG were used for the calculation of the general methylation level of each region.

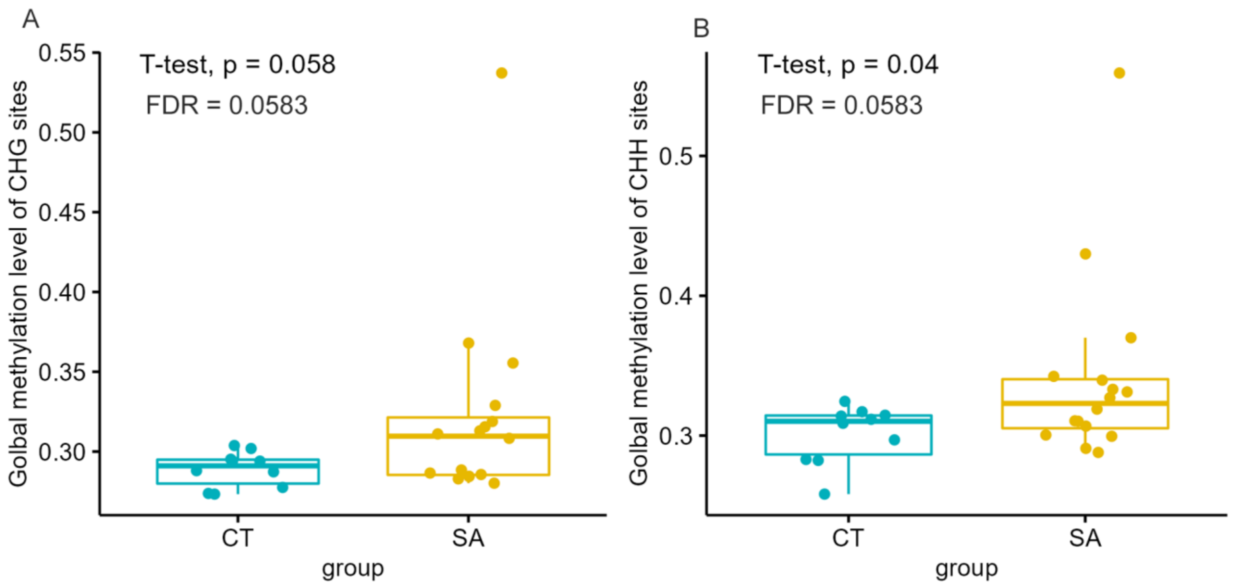

Supplemental Figure S4 Comparison of global methylation level of cytosines in the context of CHG (**A**) and CHH (**B**) between SAP and HC group.

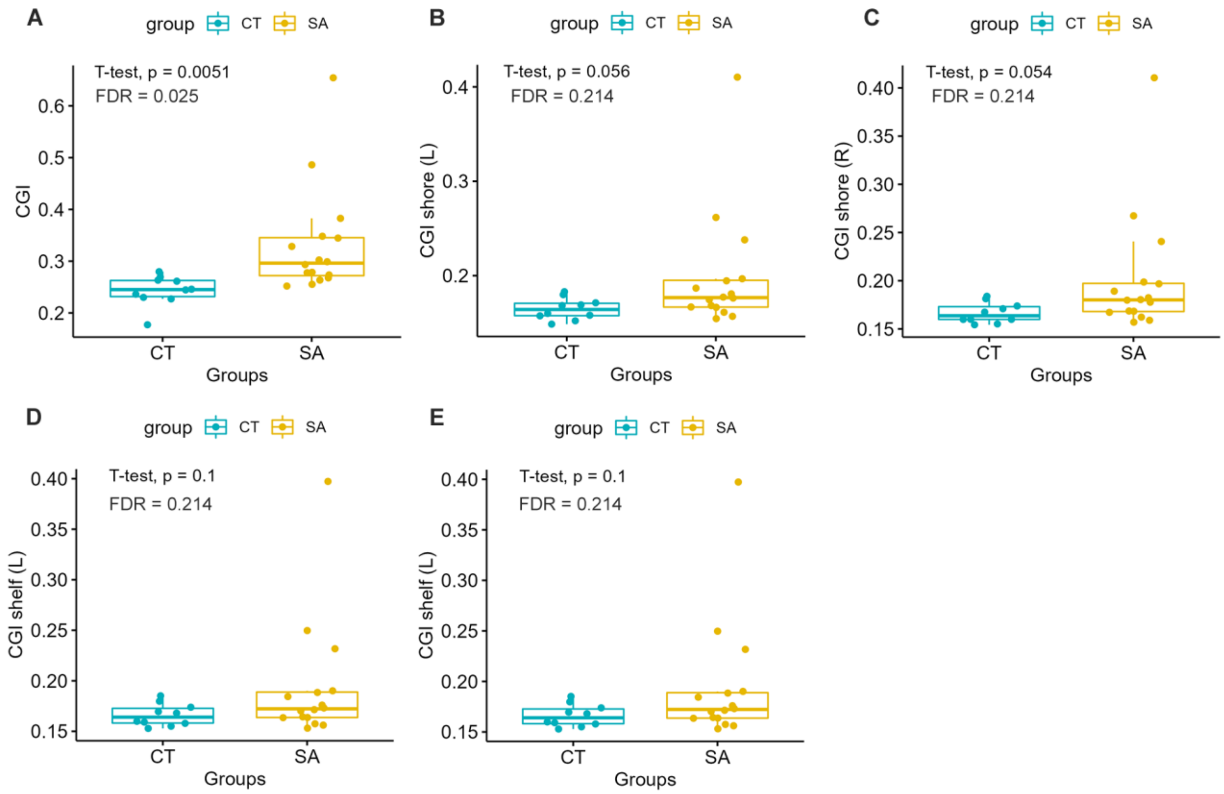

Supplemental Figure S5 Comparison of global methylation level of cytosines in the context of CHG located in CpG islands (CGI), shores and shelves between SAP and HC groups. **A-E** represent the global methylation level of CGI, left CGI shore, right CGI shore, left CGI shelf and right CGI shelf, respectively.

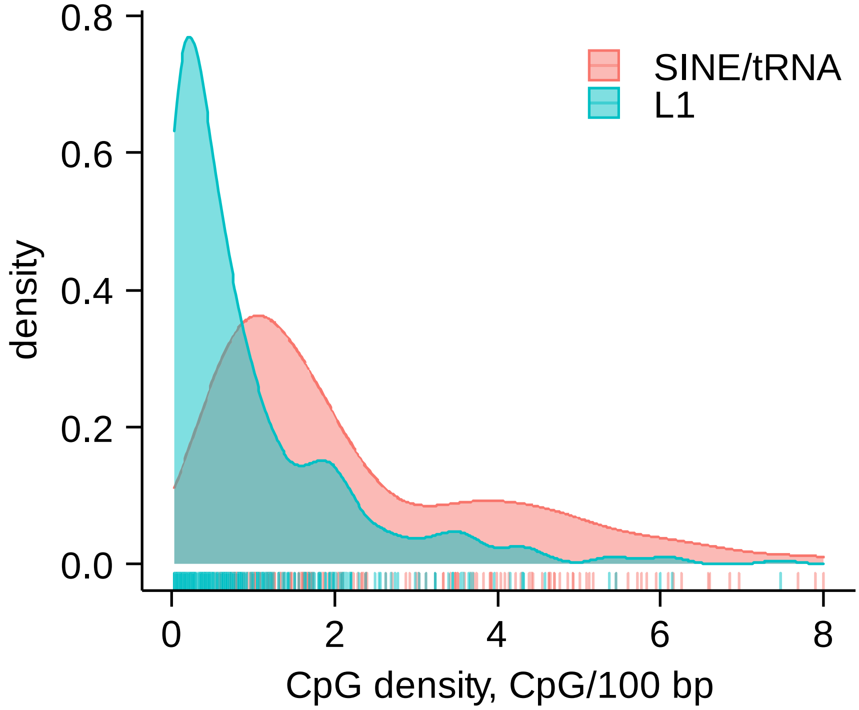

Supplemental Figure S6 Density of CpG sites in LINE-1 and t-RNA-derived SINEs.

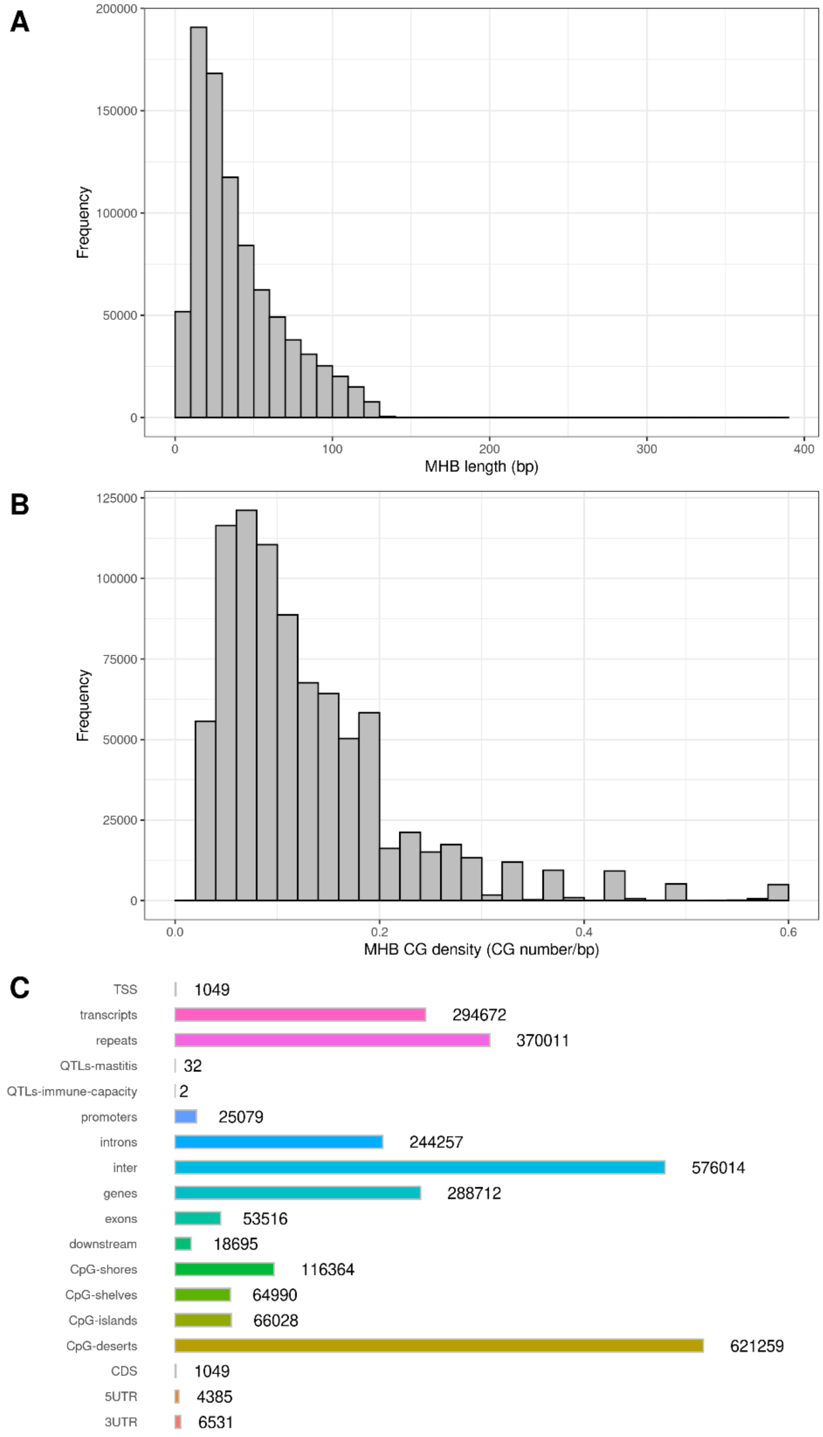

Supplemental Figure S7 Summary of identified methylation haplotype blocks (MHBs). **A** Distribution of length of MHB. **B** CpG density (number of CpG sites per bp in a MHB) distribution of MHBs. **C** Co-localization of MHBs with known genetic regions. **D** Heat-map showing the methylation status of top 50 most variable differential MHBs.

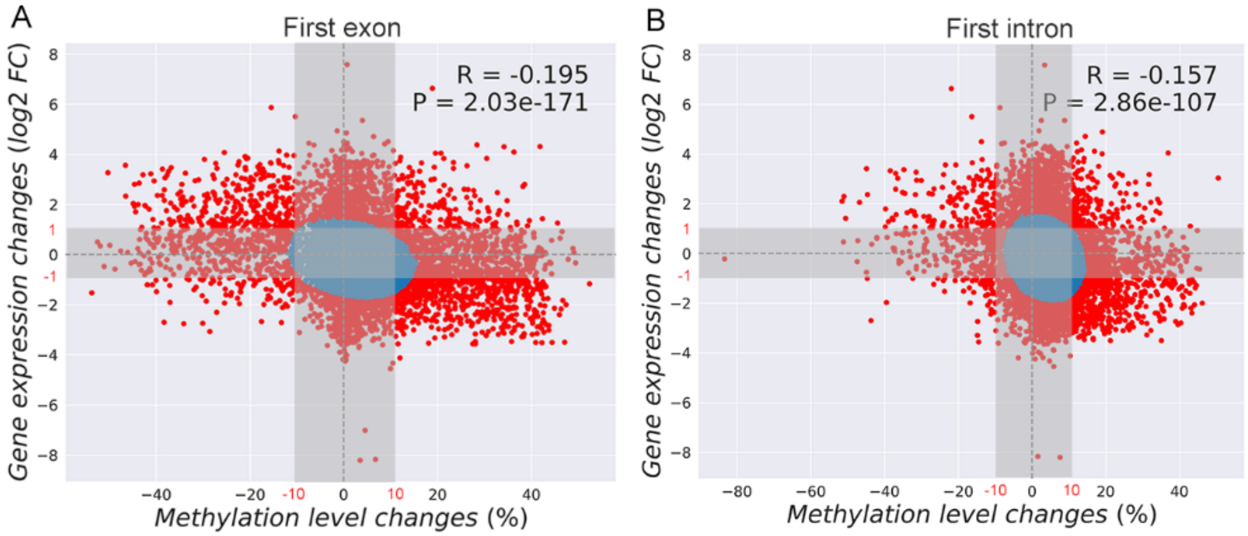

Supplemental Figure S8 Identification of differential genes with significant changes in their gene expression level and the general methylation level of first exon (**A**) and first intron (**B**). R: Pearson correlation coefficient between gene expression and methylation level of corresponding region at genome-wide, P: *P* value for the Pearson correlation coefficient.

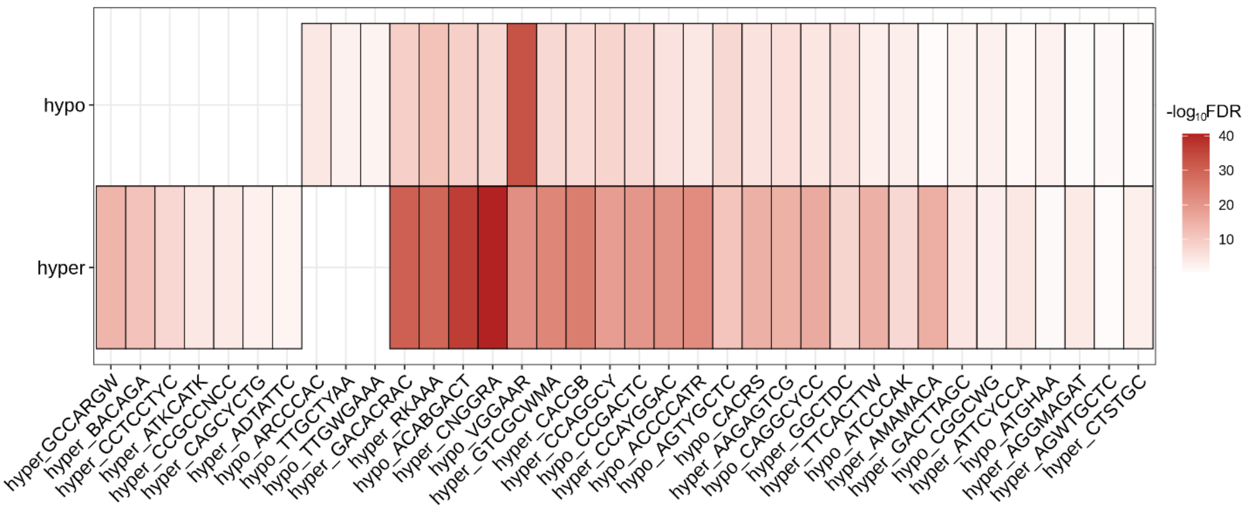

Supplemental Figure S9 *de novo* identified motifs in GE-dMHBs

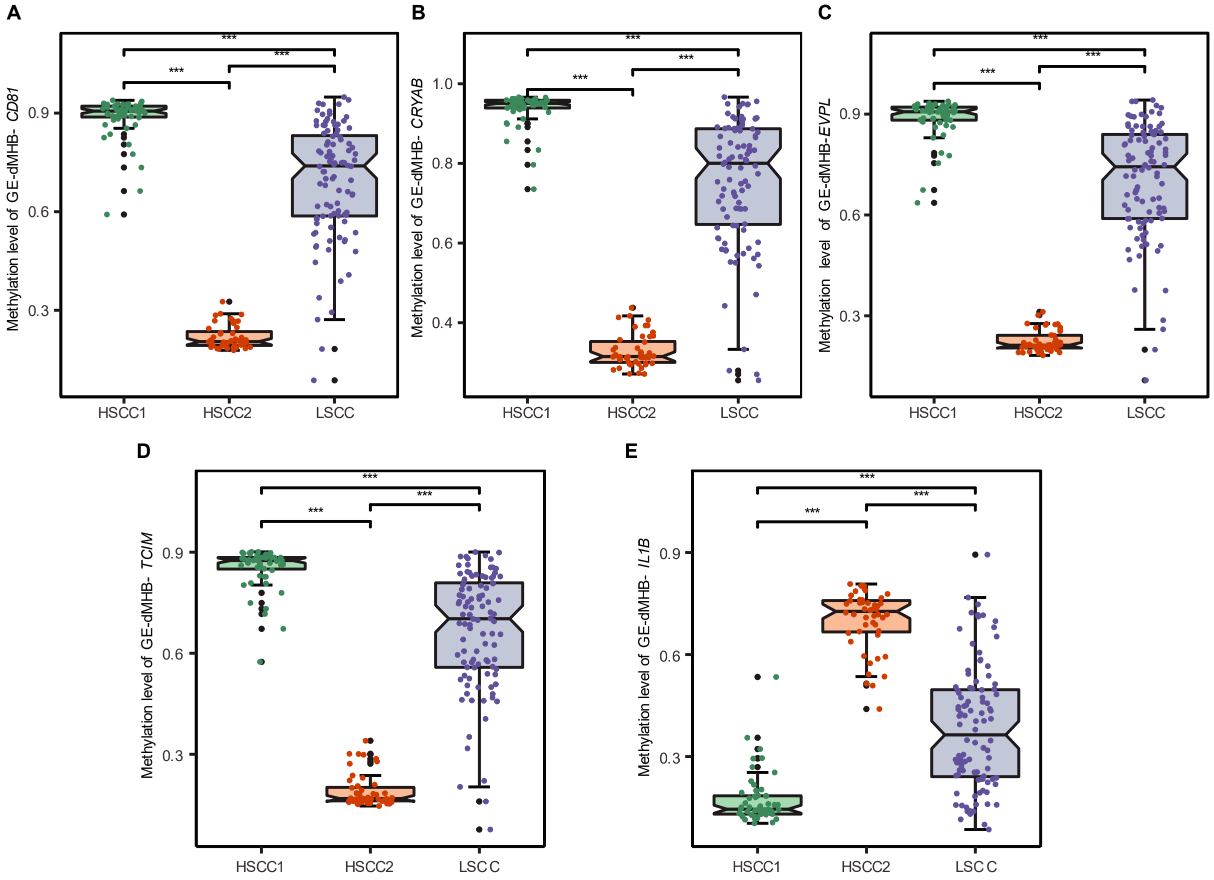

Supplemental Figure S10 Boxplots showing the methylation difference of GE-dMHBs between cows with high or low milk somatic cell count (SCC). HSCC1: the first sub-group of cows with high SCC, HSCC2: the second sub-group of cows with high SCC, LSCC: group of cows with low SCC. “***”: significant difference between corresponding groups (FDR < 0.05)

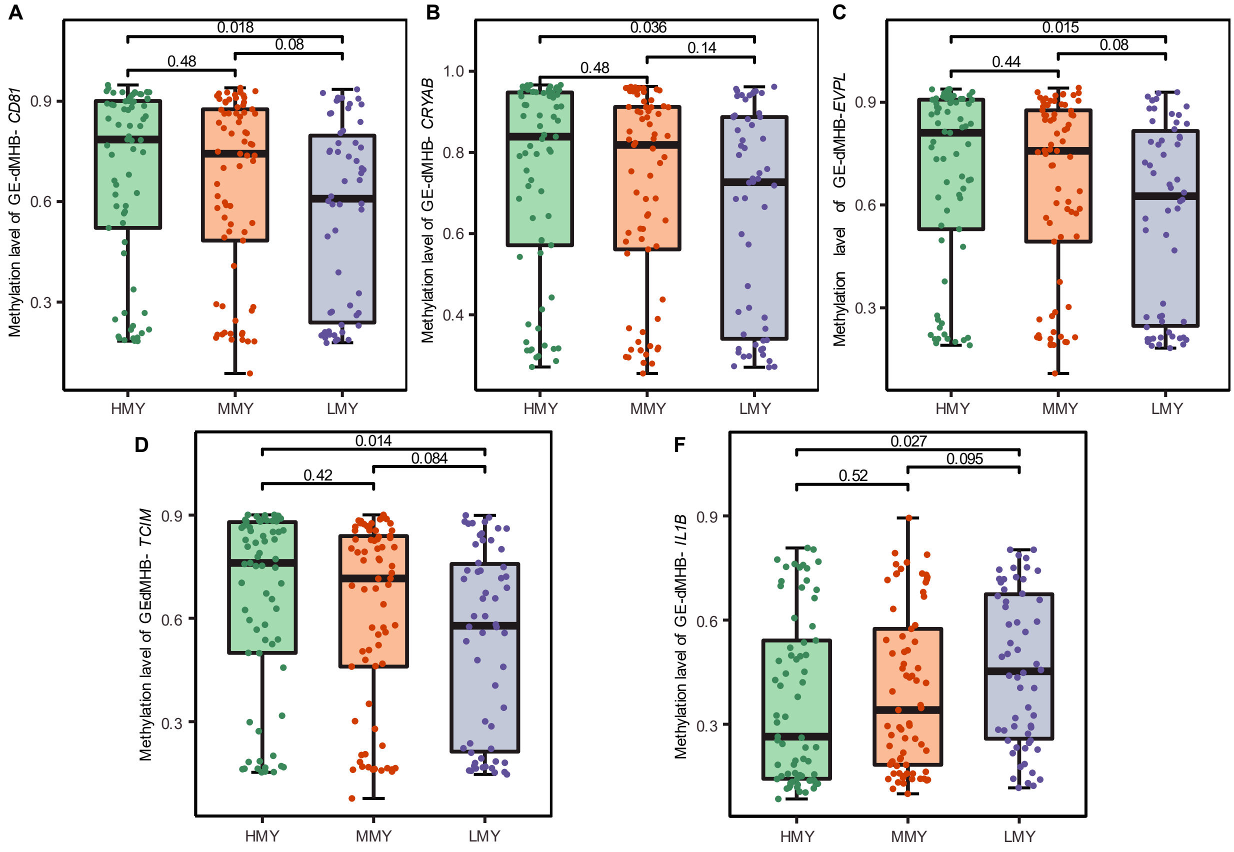

Supplemental Figure S11 Boxplots showing the methylation difference of GE-dMHBs between cows with high or low milk yield (MY). HMY: group of cows with high milk yield (> 40 kg/day), MMY: group of cows with middle level of milk yield (30~40 kg/day), LMY: group of cows with low milk yield (<30 kg/day). The number above the short bar located on the top of each boxplot represents the significance (FDR) of methylation difference between corresponding groups.
